# Improvement of Ultrasound-Assisted Enzymatic Treatment and Exogenous Amino Acids on Structural, Antioxidant, and Instrumental Taste Profile of Maillard Reaction Products from Potato Protein Hydrolysates

**DOI:** 10.3390/foods15183300

**Published:** 2026-09-18

**Authors:** Hui Liu, Miao Zhang, Taihua Mu, Hongnan Sun

**Affiliations:** 1 Laboratory of Food Chemistry and Nutrition Science, Institute of Food Science and Technology, Chinese Academy of Agricultural Sciences, Key Laboratory of Agro-Products Processing, Ministry of Agriculture and Rural Affairs, No. 2 Yuan Ming Yuan West Road, Haidian District, Beijing 100193, Chinazhangmiao@caas.cn (M.Z.); mutaihua@126.com (T.M.); 2 College of Food Science and Technology, Yunnan Agricultural University, Kunming 650201, China

**Keywords:** ultrasound, potato protein hydrolysates, exogenous amino acids, Maillard reaction, peptide structure, flavor

## Abstract

Potato protein hydrolysates (PPHs) produced by ultrasound-assisted enzymatic hydrolysis exhibit strong antioxidant activity. However, their inherent bitterness limits their application in food sectors. Therefore, this research investigated the effects of energy-divergent ultrasound (EDU)- and energy-gathered ultrasound (EGU)-assisted enzymatic treatment and exogenous amino acids (alanine (Ala), phenylalanine (Phe), and valine (Val)) on the peptide structure, antioxidant activity, and instrumental taste profile of Maillard reaction products (MRPs) prepared from PPHs. It was found that EDU and EGU significantly promoted the progress of the Maillard reaction (MR). All MRPs presented remarkable changes in the peptide structure compared to their PPH counterparts. Under the equal-mass addition of exogenous amino acids, MRPs from EDU with Ala at 120 min (EDU-Ala-120) presented the highest percentage of molecular weight (MW) <2 kDa fractions (68.55%), and a markedly improved oxygen radical absorption capacity value of 108.44 µg Trolox equivalent (TE)/mL. MRPs from EDU revealed significantly stronger umami taste and lower bitterness, while MRPs from EGU exhibited stronger sweetness. The umami taste of the MRPs was strongly negatively correlated with bitterness (−0.9517, *p* < 0.001) and positively correlated with esters (0.7293, *p* < 0.01), whereas the sweetness of the MRPs was positively associated with specific volatile components but negatively correlated with <1 kDa peptides (−0.6039, *p* < 0.05). Thus, the EDU-assisted enzymatic treatment and MR with the addition of appropriate exogenous amino acids could be a prospective way to produce natural flavor with enhanced antioxidant activity.

## 1. Introduction

Ultrasound, an emerging environmentally friendly processing technique, involves the use of sound waves with an inaudible frequency to enhance the process efficiency, improve the product quality, and extend the shelf life of food [1]. There are two kinds of ultrasonic treatment based on their energy types, energy-divergent (EDU) and energy-gathered ultrasound (EGU), which are generated by an ultrasound bath and an ultrasound probe, respectively [2]. Both EDU and EGU can significantly improve the enzymatic efficiency of protein hydrolysis, and promote antioxidant peptide production through cavitation effects, as well as stronger pressure and shear stress [3,4]. Compared with EDU, EGU is likely to generate stronger cavitation and turbulent effects, thereby promoting the exposure of more charged groups and strengthening protein–water interactions [5]. The degree of hydrolysis (DH) of potato protein hydrolysates (PPHs) and corn protein hydrolysates by EGU was higher than that by EDU [6,7]. In addition, EGU-derived PPHs and sweet potato protein hydrolysates exhibited superior antioxidant activity compared with those obtained using EDU [6,8]. This may be due to the promotion of the enzymatic hydrolysis process by EGU, thereby facilitating the release of small-molecule active peptides [9]. However, the hydrophobic amino acids those are exposed and released during enzymatic hydrolysis cause a bitter taste in antioxidant peptides, which significantly limits the application of antioxidant peptides in food [10].

During the Maillard reaction (MR), various interactions occur between the amino group of protein, peptides, or amino acids, and carbonyl groups of reducing sugars [11]. Consequently, different flavor compounds are generated, including aldehydes, ketones, furans, thiophenes, pyrazines, and pyrroles, which can mask unpleasant flavors, such as the bitter taste of antioxidant peptides [12,13]. MR can also improve the antioxidant activity of the resulting products [14]. To obtain the characteristic flavors, different exogenous amino acids can be added to the MR system, such as alanine (Ala), phenylalanine (Phe), and valine (Val), which impart floral, chocolate, and caramel notes, respectively [15,16].

Potato (*Solanum tuberosum* L.) is one of the most important crops worldwide, and China is the largest producer, contributing a quarter of the global output [17]. Potato protein is considered a high value-added food ingredient but remains underutilized, often being discharged with the waste liquid during potato starch production [18]. PPHs exhibit various biological activities, including antioxidant activities [19,20,21]. Our previous study presented that EDU- and EGU-assisted enzymatic treatments enhanced the potential antioxidant peptide productions from potato protein, as well as induced significant conformational changes in the resulting peptides [6]. However, the bitterness of these antioxidant peptides remains one of the greatest limitations to their large-scale application in the food industry [22,23]. Therefore, the production of Maillard reaction products (MRPs) from PPHs with an acceptable flavor and enhanced antioxidant activity represents a promising way for their industrial application.

Our previous work characterized antioxidant peptides from potatoes [6] and sweet potatoes [3], and examined how peptide preparation methods affect sweet-potato-peptide-derived MRPs [8]. However, the effects of exogenous amino acids on MRP formation from potato antioxidant peptides remain unknown. The purpose of this study was to examine the effects of the EDU- and EGU-assisted enzymatic treatment and exogenous amino acids (Ala, Phe, and Val) on the MR progress, peptide structure, antioxidant activity, melanoidin content, free amino acids (FAAs), instrumental taste profile, and aroma profile of MRPs from PPHs. Furthermore, the contributions of different factors (peptide structure, FAAs, and aroma profile) to the instrumental taste profile were evaluated.

## 2. Materials and Methods

### 2.1. Materials

Potato protein (72.10% purity, 6.36% moisture, 4.91% crude fiber, 3.49% ash, 1.40% reducing sugar, and 0.94% fat) was purchased from Zhuanglang Hongda Starch Processing Co., Ltd. (Pingliang, Gansu, China). Alcalase 2.4 L^®^, Neutrase 0.8 L^®^, and Flavourzyme 500 MG^®^ were purchased from Novozymes (Bagsvaerd, Denmark). Ala, Phe, and Val were purchased from Sangon Biotech (Shanghai, China). The 6-hydroxy-2,5,7,8-tetramethlchroman-2-carboxylic acid (Trolox), cytochrome c, aprotinin, vitamin B12, and glycine were obtained from Sigma-Aldrich, Inc. (St. Louis, MO, USA).

### 2.2. Fabrication of Potato Protein Hydrolysates (PPH)

PPH was fabricated using enzymatic treatment assisted by EDU and EGU as described by Liu et al. [6]. Briefly, potato protein solution (3%, w/v) was prepared by dissolving 3 g of potato protein in 100 mL of distilled water. Sequential enzymatic hydrolysis was conducted at an enzyme-to-substrate ratio of 4% (w/w). The hydrolysis was first performed with Alcalase at pH 8.0 and 50 °C for 1 h, followed by Neutrase at pH 6.0 and 50 °C for 1 h, and, finally, by Flavourzyme at pH 7.0 and 50 °C for 1 h. For EDU-assisted enzymatic hydrolysis, the reaction was conducted using an ultrasonic bath (SK8210HP, Shanghai Kudos Ultrasonic Instrument Co., Ltd., Shanghai, China) operating at a frequency of 53 kHz with a nominal power density of 40 W/L. The temperature was maintained at 50 °C during ultrasonication. For EGU-assisted enzymatic hydrolysis, the reaction was performed using a probe ultrasonic probe (Scientz-IID, Ningbo Scientz Biotechnology Co., Ltd., Ningbo, Zhejiang, China) at an amplitude of 2% with a pulse mode of 5 s on and 5 s off and a nominal power density of 40 W/L maintained at 50 °C throughout the treatment. Conventional enzymatic treatment (C) was conducted using a shaking water bath (DSHZ-300A, Taicang Experimental Equipment Factory, Suzhou, Jiangsu, China) at 70 rpm. After enzymatic reaction, each mixture was heated 15 min in boiling water, centrifuged at 10,000 *g* for 20 min at 4 °C to collect the supernatant, and then freeze-dried at −55 °C, recorded as EDU-PPH, EGU-PPH, and C-PPH, respectively.

### 2.3. Maillard Reaction Products (MRPs)

MRPs from C-PPH, EDU-PPH, or EGU-PPH were prepared according to Habinshuti et al. [8] with modification. Briefly, 0.22 g of Ala, Phe, or Val, 0.66 g of xylose, and 1.11 g of PPH were mixed with 20 mL of ultrapure water. To ensure consistency in formulation conditions across treatment groups, the same mass of each amino acid was added to each reaction. However, due to the different molecular weights of amino acids, the molar amounts of different amino acids added also varied (Ala: 2.5 mmol; Phe: 1.3 mmol; and Val: 1.9 mmol). The pH of each mixture was adjusted to 7.4, then heated at 120 °C for 30, 60, 90, and 120 min in an oil bath with magnetic stirring to prepare MRPs.

### 2.4. Browning Degree

Browning degree of PPH or MRPs was measured using a U-3010 spectrophotometer (Hitachi, Tokyo, Japan) according to the method of Yi et al. [24] with modification. The absorbance of colorless intermediate compounds of MRPs were measured at 294 nm, while that of the brown polymers formed at the advanced stage was measured at 420 nm.

### 2.5. Electronic Tongue

An electronic tongue (Alpha MOS, Toulouse, France) was used to evaluate instrumental taste profile of PPHs and MRPs with the following sensors: bitter, sour, sweet, umami, and salty.

### 2.6. Fluorescence Spectra

The fluorescence spectra of each PPH and MRP were measured with a fluorescence spectrophotometer (Hitachi, Tokyo, Japan) with an excitation wavelength of 347 nm and an emission range of 360–600 nm.

### 2.7. Fourier Transform Infrared Spectroscopy (FTIR)

The FTIR spectrum was determined using an FTIR spectrometer (Tensor II, Bruker, Billerica, MA, USA) coupled with an ATR Dura sample II accessory according to Hu et al. [25] with modification. Briefly, 1 mg of lyophilized PPH or MRP was thoroughly ground and mixed with 100 mg of dry potassium bromide powder for tableting. FTIR spectrum was then recorded as the average of 60 scans from 500 to 4000 cm^−1^.

### 2.8. Oxygen Radical Absorption Capacity (ORAC)

ORAC values of PPH or MRPs were measured according to Liu et al. [6]. Briefly, 20 μL of PPH or MRP solution (1 mg protein/mL phosphate-buffered saline (PBS) buffer, w/v) was mixed with 20 μL of 63 nM sodium fluorescein (in PBS buffer), and 20 μL of PBS buffer in a microplate, mixed gently by shaking, and then placed in the dark for 10 min at 37 °C. Subsequently, 140 μL 2,2′-azobis(2-amidinopropane) dihydrochloride (AAPH) solution (18.28 mM) was added, and the fluorescence was measured using a Fluoroskan Ascent microplate reader (Thermo Electron Corporation, Vantaa, Finland) with excitation and emission filters at 485 and 520 nm, respectively, every 2 min for a total of 60 measurements, with automatic shaking prior to the first reading. The unit of ORAC is Trolox equivalent (μg TE)/mL.

### 2.9. Melanoidins Determination

Melanoidins content of the MRPs was determined following the description by Quan et al. [26] with modification. Each MRPs was diluted 25 times and measured at 470 nm by a spectrophotometer (U-3010, Hitachi, Tokyo, Japan). Lambert–Beer equation was used for quantification with extinction coefficient of 282 L/M·cm as follows:

c=A/εb

where c is the melanoidin concentration (mmol/L MRPs), A is the absorbance, ε is extinction coefficient, and b is cuvette thickness (1 cm).

### 2.10. Molecular Weight (MW) Distribution

MW distribution was determined according to Wei et al. [27] with modification. Firstly, 1 mg/mL of PPH or MRP was prepared in 50 mM phosphate buffer (pH 7.0) with 0.15 M of NaCl, filtered using 0.22 µm membrane, and then detected at 215 nm using HPLC (Shimadzu, Kyoto, Japan) with a Superdex 30 Increase 10/300 GL column (GE healthcare Bio-Sciences AB, Uppsala, Sweden) at a flow rate of 0.5 mL/min.

### 2.11. FAA

FAA content was determined according to Wei et al. [27] with modification. A portion of 0.5 g PPH or MRPs and 4% sulfosalicylic acid was mixed, centrifuged at 10,000 *g* for 20 min, and filtered through a 0.22 μm filter. FAA content was performed by using Amino Acid Automatic Analyzer (Hitachi Ltd., Tokyo, Japan).

### 2.12. Volatile Compounds

Volatile compounds were analyzed using GC-MS (Shimadzu Corp, Kyoto, Japan). Firstly, 5 mL of PPH or MRP was added into a vial with screw cap, and equilibrated for 30 min at 50 °C. Next, volatile compounds were collected by headspace-solid-phase microextraction and then transferred to the GC injector port (Agilent Technologies, Santa Clara, CA, USA) at 250 °C for 3 min in splitless mode. Each sample was then separated by a capillary column (J&W Scientific, Folsom, CA, USA) at a total ion current 40–450 amu, carrier gas helium, and flow rate 1.8 mL/min. All volatile compounds are reported as relative peak area (%).

### 2.13. Statistical Analysis

Each experiment was performed in triplicate, and the data were presented as mean ± SD. One-way analysis of variance (ANOVA), Duncan’s multiple-comparison test, and Pearson correlations were analyzed using SPSS 27.0 (IBM Corporation, Armonk, NY, USA). Graphics were generated using OriginPro8 (Origin Lab Corporation, Northampton, MA, USA). Significant difference was set at *p* < 0.05.

## 3. Results and Discussion

### 3.1. The pH Values and Browning Intensity

The pH values and browning intensity of PPHs and their MRPs are shown in Table S1 and Table 1. As shown in Table S1, the pH decreased progressively throughout the 120 min heating period; absorbances at 294 and 420 nm values increased strictly in a time-dependent manner from 30 to 120 min, indicating the continuous formation of both intermediate and advanced Maillard reaction products over time.

In the case of Ala, pH values of the MRPs decreased from 7.40 (C-PPH, EDU-PPH, and EGU-PPH) to 5.47 (C-Ala-120), 5.48 (EDU-Ala-120), and 5.41 (EGU-Ala-120), respectively (Table 1). For Phe and Val, the pH values decreased to 5.65 and 5.30 (C-Phe-120 and C-Val-120), 5.34 and 5.40 (EDU-Phe-120 and EDU-Val-120), and 5.38 and 5.34 (EGU-Phe-120 and EGU-Val-120), respectively. The decrease in pH was attributed to the generation of formic acid, acetic acid, and free carboxyl groups, as well as the amino groups’ consumption of the peptides [14].

Higher absorbances at 294 and 420 nm indicate higher contents of colorless intermediate compounds and advanced-stage brown polymers, respectively [14]. For Ala, the absorbance at 294 nm of EDU-Ala-120 (1.84) was the highest, followed by EGU-Ala-120 (1.66) and C-Ala-120 (1.53), respectively (*p* < 0.05, Table 1), suggesting the formation of more intermediate compounds in EDU-Ala-120. For Phe, the absorbance at 294 nm of EGU-Phe-120 (2.12) was the highest, followed by EDU-Phe-120 (1.84) (*p* < 0.05). For Val, the absorbance at 294 nm of EGU-Val-120 (2.31) was the highest, followed by EDU-Val-120 (1.93) (*p* < 0.05). The absorbance at 420 nm exhibited similar increasing trends (Table 1), revealing the generation of more brown compounds. Under the equal-mass addition of exogenous amino acids, the absorbance at 420 nm of EGU-Val-120 (0.82) was the highest among all MRPs, followed by EGU-Phe-120 (0.72), C-Val-120 (0.65), EDU-Val-120 (0.63), and EDU-Phe-120 (0.61) (*p* < 0.05, Table 1). It was reported that a higher absorbance at 420 nm was attributed to peptide cross-linking, which led to the generation of more brown substances at the final stage of the MR [27]. Notably, MRPs with EDU and EGU had a higher absorbance at 294 and 420 nm (Table 1, *p* < 0.05), indicating that EDU and EGU promoted the formation of intermediate and final compounds in MRPs. Corzo-Martínez et al. [28] and Guo et al. [29] also reported that ultrasonic treatment increased the levels of intermediate and final compounds during the MR. It should be noted that the potato protein contained 1.40% reducing sugars, which may react with amino groups from PPHs and amino acids, promoting Maillard reactions.

Moreover, it is also worth noting that the amino acids were added in equal masses rather than same molar amounts. Thus, the comparisons in this study reflect the overall impact of different amino acids under the same processing conditions, rather than directly comparing their intrinsic Maillard reactivity under equimolar conditions. This difference needs careful consideration when interpreting the comparison results of the three amino acids. Although Ala had the highest molar concentration (2.5 mmol), the MRPs of EGU-Val-120 exhibited the highest A_294_ and A_420_ values among all samples, respectively. It suggests that the Maillard reaction is not entirely dependent on the molar amount of the amino group, while the structure difference of each amino acid may also play an important role in the formation of intermediates and the advanced-stage product. However, the comparisons between amino acids in this study primarily reflect the combined effects of their concentration and inherent reactivity. Future research should employ equimolar addition methods to analyze the specific contribution of a single variable to MRP formation.

### 3.2. Intrinsic Fluorescence

**Figure 1:**
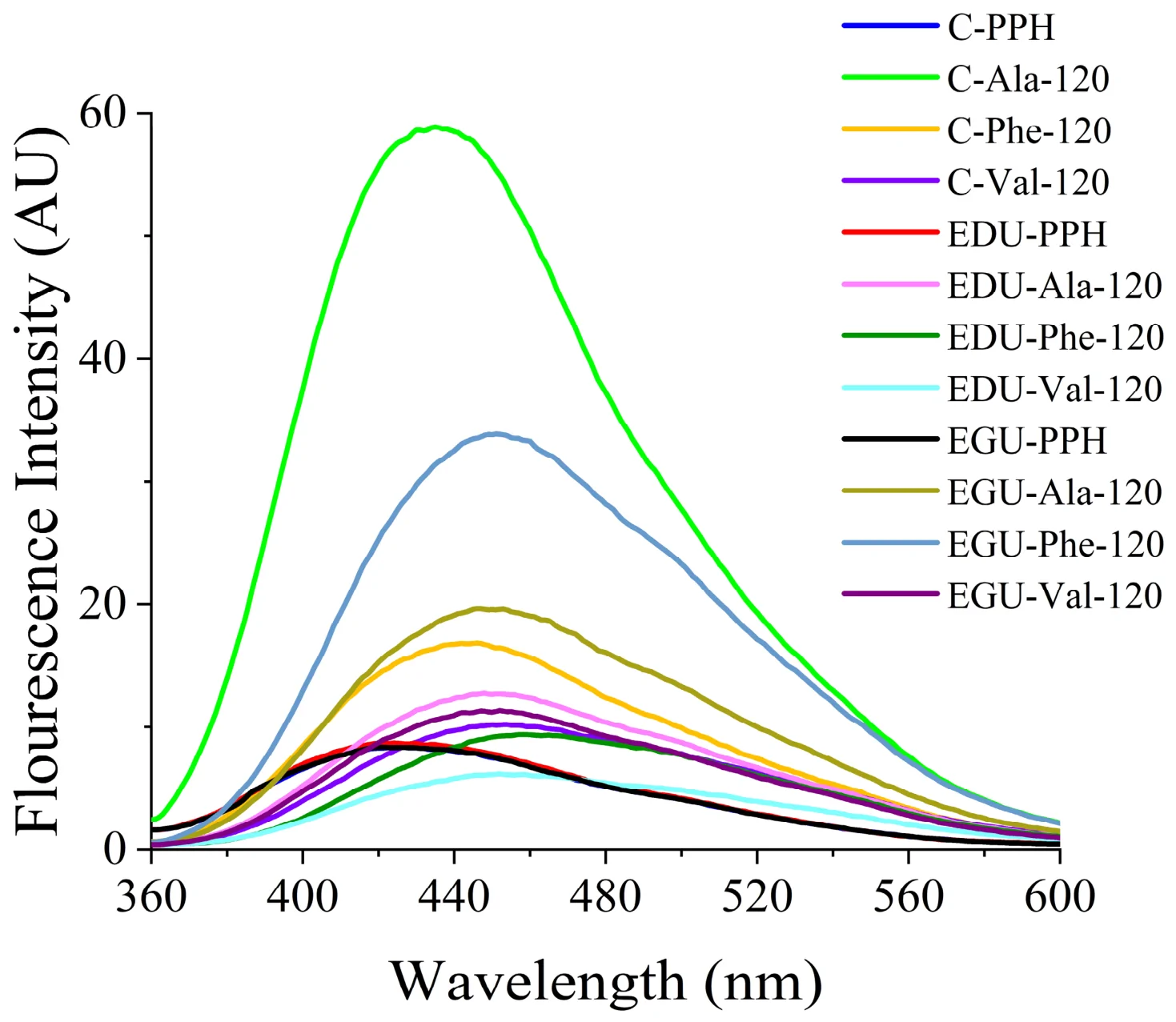
Fluorescent intensity of potato protein hydrolysates and their Maillard reaction products. C, non-ultrasonicated conventional enzymatic hydrolysis; EDU, energy-divergent ultrasound; EGU, energy-gathered ultrasound. Ala, Alanine; Phe, Phenylalanine; Val, Valine.

The intrinsic fluorescence of PPHs and MRPs is shown in Figure 1, which is considered an indicator of the MR progress. During the progress of the MR, small-molecule fluorescent substances such as imidazole and pyrrole are generated as precursors of brown compounds [30]. In addition, C-PPH, EDU-PPH, and EGU-PPH also showed a weak fluorescence intensity (Figure 1), which was attributed to the generation of fluorescent substances from heating during enzymatic hydrolysis [8].

Under the equal-mass addition of exogenous amino acids, C-Ala-120 exhibited the highest fluorescence intensity at 58.92 among C-MRPs, followed by C-Phe-120 (16.84) and C-Val-120 (10.21). For EDU-MRPs, the fluorescence intensity variation trend was the same for all C-MRPs with the addition of the same amino acid. EDU-Ala-120, EDU-Phe-120, and EDU-Val-120 had fluorescence intensities of 12.76, 9.37, and 6.17, respectively (Figure 1). Furthermore, EGU-Phe-120 had a higher fluorescence intensity (33.85) than EGU-Ala-120 (19.65) and EGU-Val-120 (11.35). The differences in fluorescence intensity may be related to the extent of the MR progression, as Amadori compounds, stably formed via the intramolecular rearrangement of Schiff bases, subsequently undergo dehydration and fission to yield fluorescent substances [31].

Compared to all PPHs that did not undergo the MR, the emission wavelengths of all MRPs showed a red-shift from 422–426 nm to 435–458 nm. With the addition of Ala, the emission wavelengths were 435 nm for C-Ala-120, 448 nm for EDU-Ala-120, and 447 nm for EGU-Ala-120, respectively. In the case of Phe, the emission wavelengths were 446 nm for C-Phe-120, 458 nm for EDU-Phe-120, and 451 nm for EGU-Phe-120, respectively. While for Val, all MRPs exhibited similar emission wavelengths, which were 453 nm for C-Val-120, 452 nm for EDU-Val-120, and 452 nm for EGU-Val-120. During MR, the fluorescence spectrum changes significantly due to the generation of MRPs [32]. The above results suggested that EDU and EGU, with the addition of Ala or Phe, promoted the MR process and the formation of MRPs with more complex structures.

### 3.3. Peptide Structures

**Figure 2:**
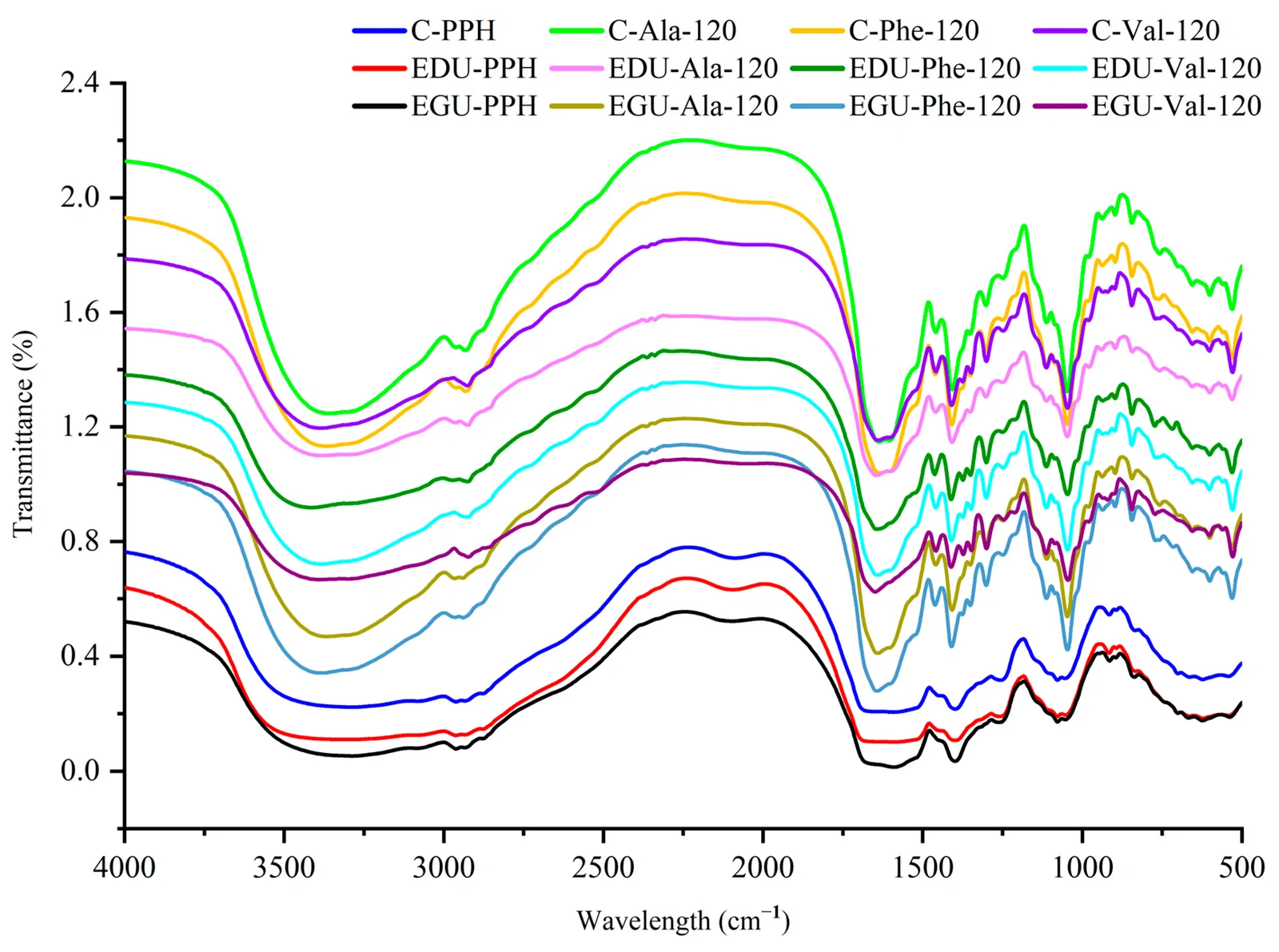
FTIR of potato protein hydrolysates and their Maillard reaction products. C, non-ultrasonicated conventional enzymatic hydrolysis; EDU, energy-divergent ultrasound; EGU, energy-gathered ultrasound. Ala, Alanine; Phe, Phenylalanine; Val, Valine.

The peptide structures of PPHs and MRPs as revealed by FTIR spectroscopy are shown in Figure 2. The peaks at 1600–1700, 1530–1550, and 1200–1300 cm^−1^ represent the C=O stretching vibration of the amide I band, the N-H stretching and C-N deformation of the amide II band, and the C-N stretching and N-H-bending of the amide III band, respectively [33]. Peaks at 3237–3301 stand for the N-H vibration of amide A, while another peak at 2914–2939 cm^−1^ reflects the C-H stretching of amide B [34].

The major peak in the region of the amide A of PPHs was at 3286 cm^−1^, which shifted to 3365 cm^−1^ after MR (Figure 2). This shift is attributed to the formation of O-H stretching from the hydroxyl group of the peptide and N-H stretching in amide A after MR. Shift changes also occurred in the C-C, C-O, and C-N regions and amide bands (I, II, and III) (Figure 2). These shifts were related to the loss of certain functional groups during MR, especially –NH_2_ in amino acids, which led to the generation of new functional groups in MRPs, such as C=O in Amadori rearrangement compounds and C=N in pyrazines and Schiff bases [35]. The absorption intensities of C-H stretching in MRPs with Val at 2914–2939 cm^−1^ were higher than those of all PPHs and MRPs with Ala and Phe, an observation that was due to the exposed C-H bond in Val as compared to Ala or Phe. The absorption bands of all MRPs in the 1050–900 cm^−1^ were significantly stronger than those of all PPHs, suggesting stronger side chain vibrations indicative of changes in the peptide structure. In addition, new absorption bands in the Amide III, C-C, C-O, and C-N regions of the MRP spectra could be attributed to Strecker degradation [8]. Furthermore, the intensity of the amide A and amide B regions (3380–3340 cm^−1^) decreased in EDU- and EGU-MRPs, suggesting a decrease in free –OH groups. It was reported that ultrasound could promote the cross-linking of free hydroxyl group of peptides upon MR [36]. Moreover, EDU-MRPs and EGU-MRPs presented a weaker transmittance intensity in the amide I and II regions. This decrease was due to the formation of covalent bonds between carbonyl groups and amino acid residues [37]. A reduction in transmittance intensities for C-O stretching, C-N stretching, N-H stretching, and –OH stretching was observed in EDU-MRPs and EGU-MRPs compared with C-MRPs (Figure 2). This indicates that ultrasound can promote a greater degree of cross-linking in ultrasound-assisted enzymatic peptides [8].

### 3.4. MW Distribution

**Figure 3:**
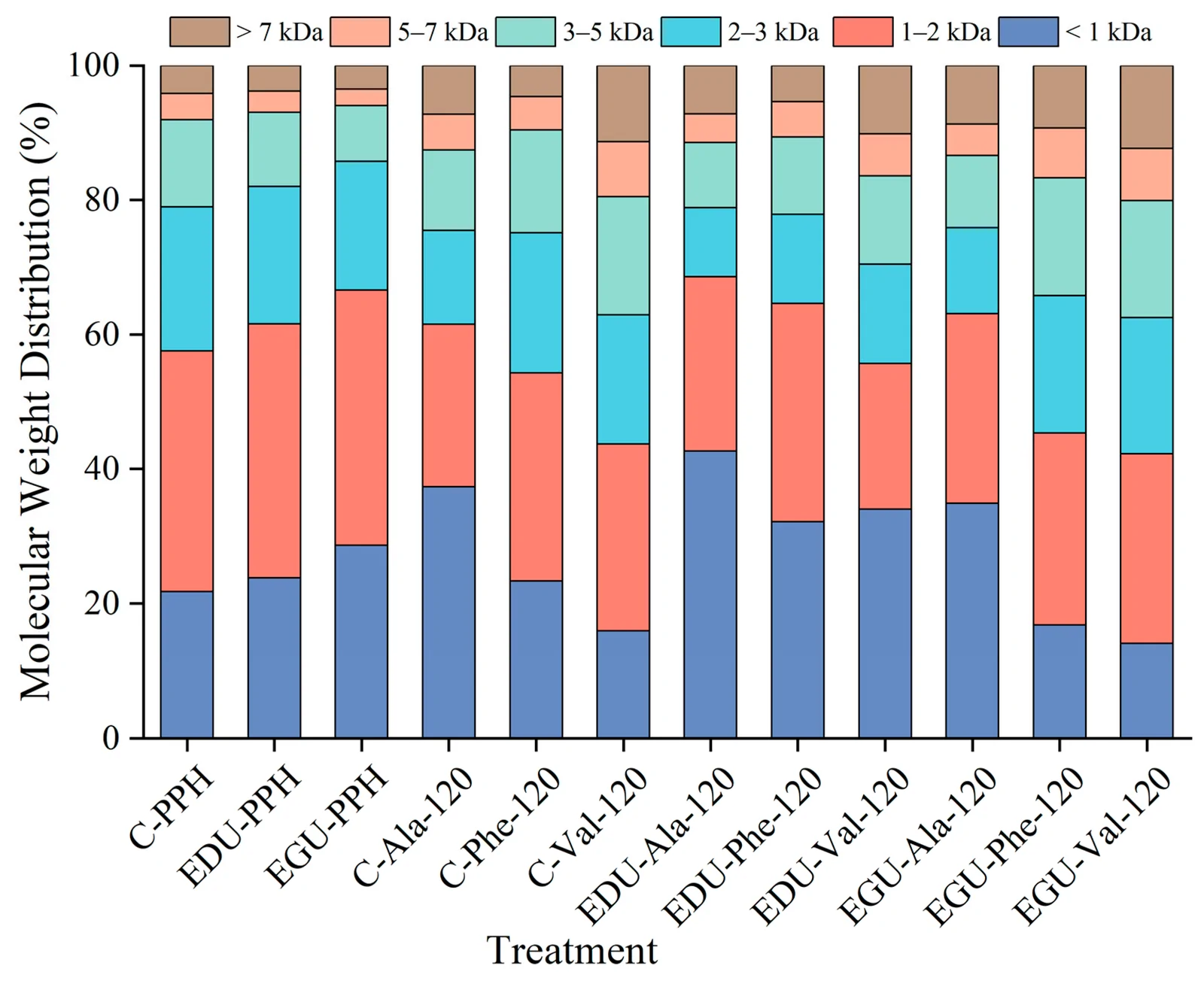
Molecular weight distribution of potato protein hydrolysates and their Maillard reaction products. C, non-ultrasonicated conventional enzymatic hydrolysis; EDU, energy-divergent ultrasound; EGU, energy-gathered ultrasound. Ala, Alanine; Phe, Phenylalanine; Val, Valine. 120, Maillard reaction time 120 min.

The MW distributions of PPHs and MRPs are shown in Figure 3. Compared with C-PPH (57.55%), the proportions of MW <2 kDa fractions in EDU-PPH and EGU-PPH increased significantly to 61.59% and 66.58% separately, whereas MW >7 kDa fractions of EDU-PPH and EGU-PPH decreased to 3.98% and 3.54% as compared with C-PPH (4.21%, Figure 3). This can be explained by the fact that EDU- and EGU-assisted enzymatic hydrolysis were more favorable for producing low MW fractions [8].

After MR, EDU-MRPs contained the highest proportions of MW <2 kDa, which were 68.55%, 64.59%, and 55.65% for EDU-Ala-120, EDU-Phe-120, and EDU-Val-120, respectively (*p* < 0.05, Figure 3). In contrast, EGU-MRPs had the most fractions with MW >7 kDa, which were 8.72%, 9.30%, and 12.34% for EGU-Ala-120, EGU-Phe-120, and EGU-Val-120, respectively (Figure 3), suggesting more peptide cross-linking [27]. Compared with the addition of Val or Phe at the same mass, the MRPs with Ala had more MW <2 kDa fractions, which were 61.51%, 68.55%, and 63.06% for C-Ala-120, EDU-Ala-120, and EGU-Ala-120, respectively (Figure 3). Thus, the higher antioxidant activity of EDU-Ala-120 was related to the generation of fractions with MW <2 kDa, due to the increased possibility of free radical scavenging interactions and/or electron donation by these low MW peptides [38].

### 3.5. FAA

Regarding the total FAA (TFAA), EDU-PPH and EGU-PPH exhibited a slightly higher TFAA content (8.55 and 8.43 g/100 g) than C-PPH (8.33 g/100 g), but no significant differences (*p* > 0.05, Table S2). The TFAA contents of EDU-MRPs with Ala, Phe, or Val were lower than those of C-MRPs (*p* < 0.05), while EGU-Ala-120 (16.39 g/100 g) showed a higher TFAA, followed by EGU-Val-120 (16.07 g/100 g, *p* < 0.05). This phenomenon might be due to both the Strecker and thermal degradation of amino acids that occurred during MR [39]. Ultrasonic treatment could generate free radicals which would oxidize the amino groups of amino acids, resulting in a highly reactive aldehyde formation, a process that was favorable for the MR process [8].

### 3.6. ORAC and Melanoidins

**Figure 4:**
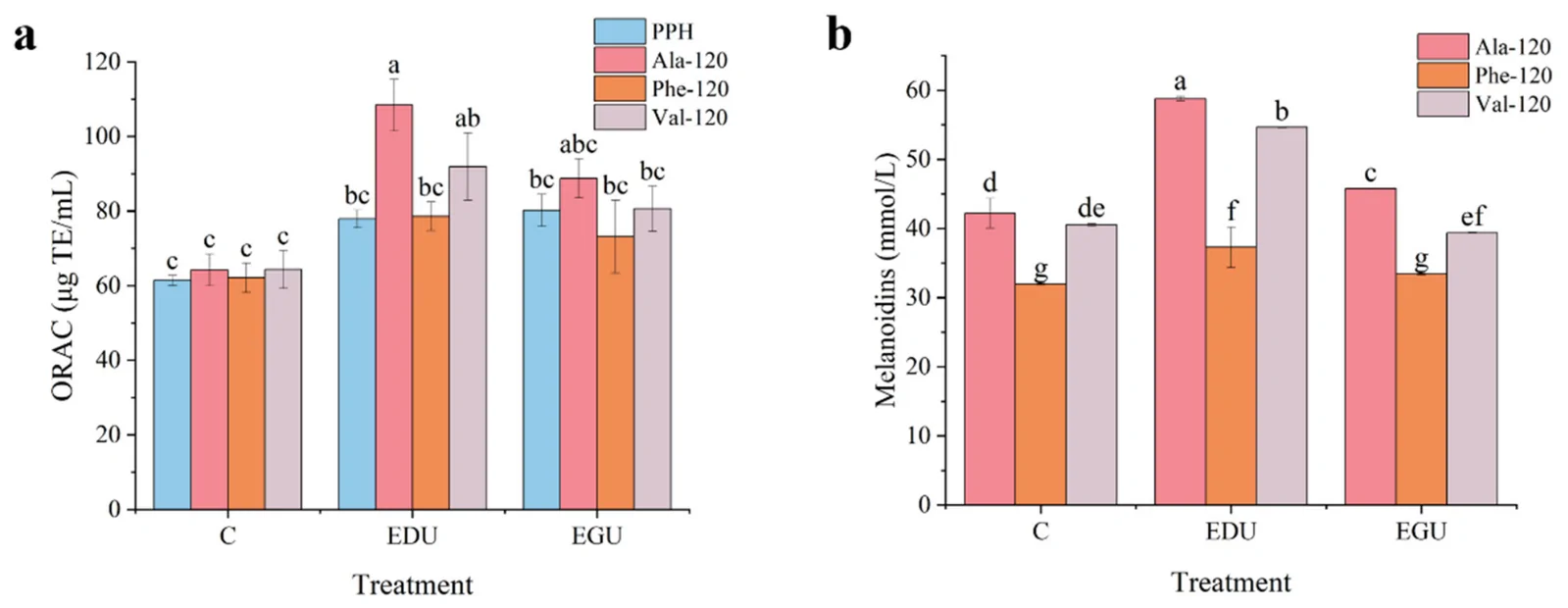
Oxygen radical absorbance capacity (ORAC) (**a**) and melanoidin contents (**b**) of potato protein hydrolysates and their Maillard reaction products. Values following different letters are significantly different (*p* < 0.05). C, non-ultrasonicated conventional enzymatic hydrolysis; EDU, energy-divergent ultrasound; EGU, energy-gathered ultrasound. Ala, Alanine; Phe, Phenylalanine; Val, Valine. 120, Maillard reaction time 120 min.

The ORAC values and melanoidin contents of PPH and MRPs are shown in Figure 4a,b. The ORAC values of C-PPH, EDU-PPH, and EGU-PPH were 61.45, 77.91, and 80.24 µg TE/mL, respectively (Figure 4a). Notably, EDU-PPH and EGU-PPH exhibited higher antioxidant activity than C-PPH (*p* < 0.05). After MR, EDU-MRPs and EGU-MRPs presented a stronger ORAC than C-MRPs (Figure 4a). Increases in the ORAC of the MRPs as compared to their hydrolysate counterparts could be due to both the breakdown of certain peptides with low antioxidant activity and melanoidin formation during the MR process [8]. Among MRPs prepared with the same exogenous amino acids of Ala, Phe, or Val at the same mass, EDU-MRPs exhibited the highest ORAC values of 108.44 (EDU-Ala-120), 78.60 (EDU-Phe-120), and 91.95 (EDU-Val-120) µg TE/mL (*p* < 0.05, Figure 4a). Obviously, EDU-Ala-120 showed the highest ORAC values among all PPHs and their MRPs (*p* < 0.05, Figure 4a). The antioxidant capacity of the MRPs was attributed to amino acid types [40]. Previous studies have also demonstrated the high antioxidant properties of ginsenoside MRPs prepared with Ala [41].

Melanoidins are late-stage compounds formed during MR, characterized by a high molecular weight and a brown color [42]. Among the MRPs prepared with different exogenous amino acids at the same mass, those containing Ala exhibited higher melanoidin contents of 42.21 (C-Ala-120), 58.77 (EDU-Ala-120), and 45.77 mmol/L (EGU-Ala-120), respectively, followed by those with Val (*p* < 0.05, Figure 4b). Notably, EDU-Ala-120 had the highest melanoidin content as compared to other MRPs (*p* < 0.05, Figure 4b), a finding that was consistent with its high ORAC value (*p* < 0.05, Figure 4a). Manzocco et al. [43] also reported that high-molecular-weight components in MRPs, such as melanoidins, were the main antioxidant products in processed foods. Ala exhibited higher ORAC values and melanoidin content across different amino acids in MRPs, which may be related to its higher molar concentration and/or unique structure. Future analyses of equimolar concentrations are needed to investigate the impact of the amino acid structure on MRPs.

### 3.7. Aromatic Compounds

In PPHs and their MRPs, 143 volatile compounds were identified (Table S3). These included 24 aldehydes, 42 ketones, 13 esters, 20 alcohols, 6 acids, 5 furans, 9 pyrazines, 2 pyrroles, 4 sulfur-containing compounds, 5 alkanes, 8 alkenes, 1 imine, and 4 other species (Table S3).

Lipid oxidation and Strecker degradation were the major aldehyde formation ways during MR. These compounds have lower odor threshold values, indicating that they are important odor-active compounds [44]. Compared with the PPH, the aldehyde contents of all MRPs were higher, and the order was EGU-Phe-120 (65.80%) > EDU-Phe-120 (58.79%) > C-Phe-120 (58.59%) > EGU-Ala-120 (55.22%) > EGU-Val-120 (46.15%) > EDU-Ala-120 (43.09%) > C-Val-120 (42.89%) > C-Ala-120 (40.11%) > EDU-Val-120 (36.80%) > C-PPH (14.27%) > EGU-PPH (12.02%) > EDU-PPH (9.24%). During heating, the resulting hydroperoxides were degraded into various volatile compounds, especially aldehydes. In addition, the formation of aldehydes via the Strecker degradation of amino acids could be enhanced by ultrasonic waves through the generation of cavitation, shear stress, and pressure [8]. Benzaldehyde, which has a cherry-like aroma [35], was the dominant aldehyde in the PPHs and their MRPs. Similar to the trend observed for total aldehyde, EDU-Phe-120 (17.04%), C-Phe-120 (15.88%), and EGU-Phe-120 (15.52%) had a much higher benzaldehyde proportion than other MRPs (Table S3). Phenylalanine could generate phenylacetaldehyde as its Strecker aldehyde, and, further, formed benzaldehyde [45].

Compared with the PPHs, the total ketone content of all MRPs was lower (*p* < 0.05, Table S3), and the order was EDU-PPH > C-PPH > EGU-PPH > EDU-Ala-120 > C-Ala-120 > EDU-Val-120 > C-Val-120 > EGU-Ala-120 > EGU-Val-120 > C-Phe-120 > EDU-Phe-120 > EGU-Phe-120 (Table S3). Generally, ketones have relatively high odor thresholds, which contributes to the better aroma profile of food products [46].

Esters impart sweet, floral, and fruity odors, which are produced via the esterification of carboxylic acids and alcohols [47]. Ethyl acetate, ethyl butyrate, 2-methyl butyrate, ethyl valerate, and methyl butyrate were the key volatile compounds identified. C-MRPs and EGU-MRPs containing Ala exhibited higher proportions of esters than those with Phe and Val, whereas EDU-MRPs with Ala or Val showed relatively high ester proportions (Table S3). Due to their low odor thresholds, even a small amount of esters can make a significant contribution to the overall aroma [48].

Similar to ketones, alcohols contribute little to food aroma due to their high odor threshold [48]. Furans are responsible for the odor perception for green bean, fruity, and buttery odor notes [49]. The proportions of furans in EDU-MRPs and EGU-MRPs were lower compared to those in their respective PPHs (Table S3). Pyrazines are characteristic compounds that impart roasted and nutty flavors [50]. Pyrazines and pyrroles were detected in all MRPs, but not in the PPHs (Table S3). Meat-flavored disulfides were produced after MR, among which EDU-Ala-120 exhibited the highest abundance (Table S3).

### 3.8. Instrumental Taste Profile

**Figure 5:**
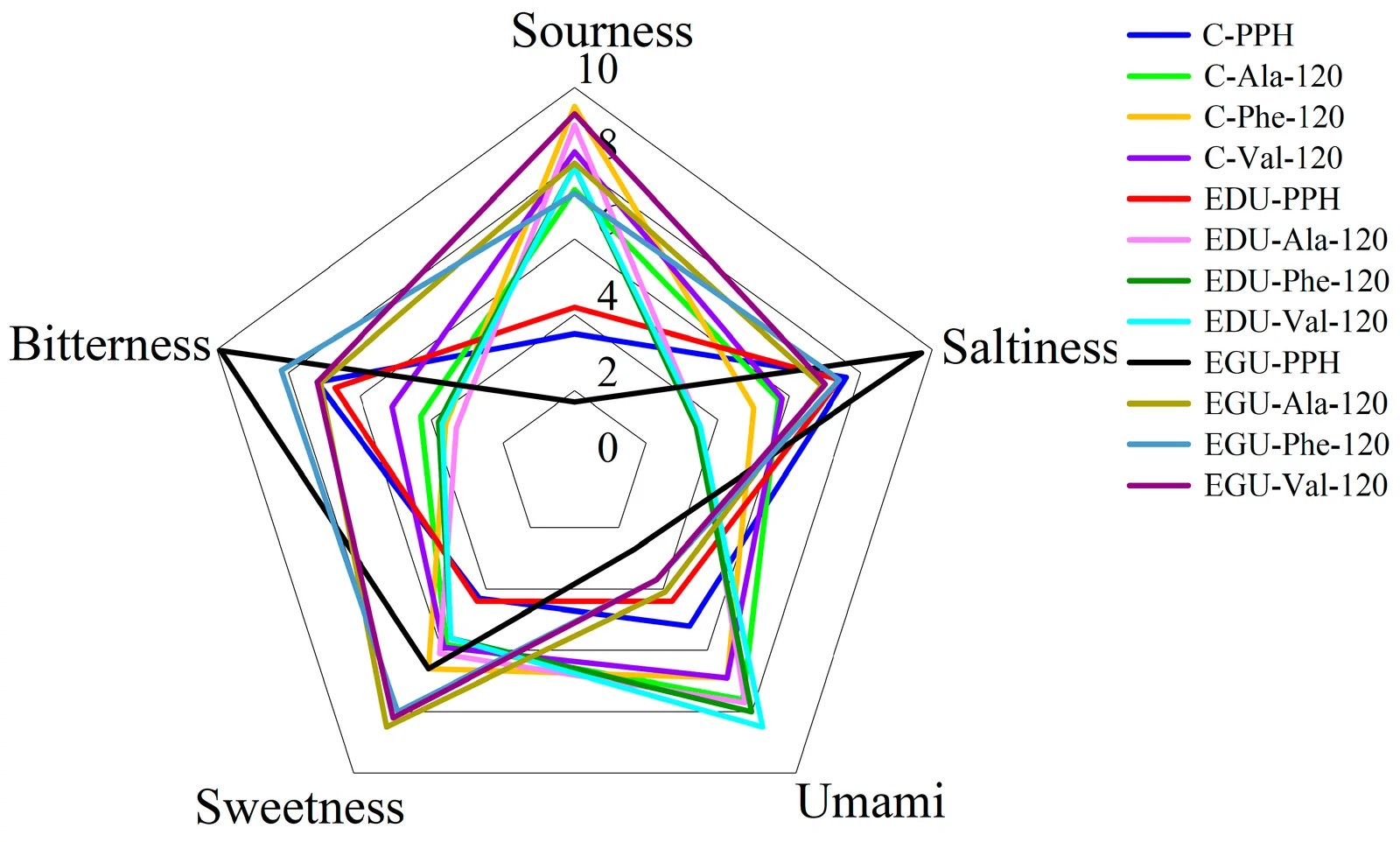
Instrumental taste profile of potato protein hydrolysates and their Maillard reaction products. C, non-ultrasonicated conventional enzymatic hydrolysis; EDU, energy-divergent ultrasound; EGU, energy-gathered ultrasound. Ala, Alanine; Phe, Phenylalanine; Val, Valine.

The instrumental taste profile of PPHs and their MRPs are shown in Figure 5. All PPHs possessed high bitterness (7.2, 6.7, and 9.9) and saltiness (7.6, 7.4, and 9.7), but low sweetness (4.3, 4.4, and 6.6), umami (5.2, 4.4, and 2.7), and sourness (3.5, 4.2, and 1.7). Among all PPHs, EGU-PPH had the highest bitterness (9.9) and saltiness (9.7), but the lowest umami taste (2.7). This might be explained by the exposure and release of more hydrophobic amino acids during hydrolysis, which was related to the high pressure and shear force generated by EGU [37].

With the progress of MR with different exogenous amino acids, the bitterness and saltiness of all MRPs were decreased, while the sweetness, umami, and sourness were increased. The reduction in bitterness after MR is attributed to the degradation of bitter peptides [51]. For C-MRPs, C-Ala-120 presented the highest umami (7.6), while C-Phe-120 had the highest sweetness (6.6). For EDU-MRPs, EDU-Ala-120 exhibited the highest sweetness (6.1) and the lowest bitterness (3.3). For EGU-MRPs, EGU-Ala-120 had the highest sweetness (8.5) and the lowest bitterness (7.1). Notably, the addition of Ala was more conducive at producing MRPs with a higher sweetness, while EDU tended to produce less bitter MRPs than EGU. Chen et al. [35] also reported that EDU resulted in a better flavor than EGU for grass carp protein hydrolysates. However, this study used electronic tongue technology, rather than a trained panel for sensory evaluation. Although this technology can effectively distinguish between differences in the taste characteristics of MRPs, it still differs from complex human flavor perception. Therefore, future assessments using a trained sensory evaluation panel are needed to validate the observed taste differences.

### 3.9. Contribution of MW Distribution, FAAs, and Aroma Compounds to Instrumental Taste Profile

**Figure 6:**
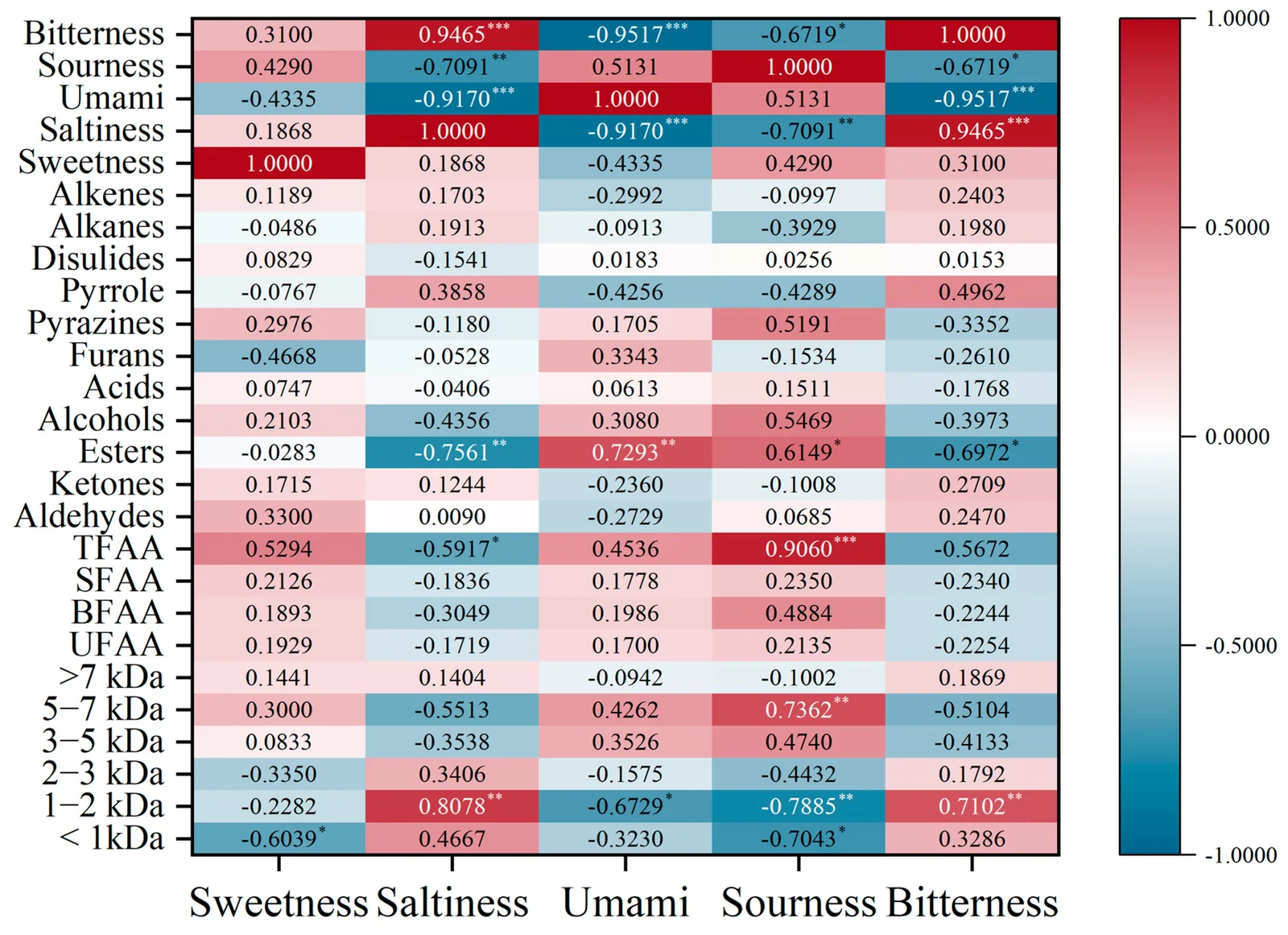
Pearson correlations between the molecular weight distribution, contents of FAAs and aroma compounds, and instrumental taste profile of PPH and their MRPs. *: *p* < 0.05; **: *p* < 0.01; ***: *p* < 0.001. The reported *p* values are uncorrected for multiple comparisons.

The correlations between the instrumental taste profile and MW distribution, FAAs, and aroma compounds are summarized in Figure 6 and Figure S1 and Table S4. For different tastes, bitterness is positively correlated with saltiness (0.9465, *p* < 0.001), while it is negatively correlated with umami (−0.9517, *p* < 0.001) and sourness (−0.6719, *p* < 0.05). MW is also an important factor affecting the instrumental taste profile; 1–2 kDa peptides are positively related to saltiness (0.8078, *p* < 0.01) and bitterness (0.7102, *p* < 0.01), but negatively related to sourness (−0.7885, *p* < 0.01) and umami (−0.6729, *p* < 0.05). The <1 kDa peptides are negatively related to sourness (−0.7043, *p* < 0.05) and sweetness (−0.6039, *p* < 0.05), while 5–7 kDa peptides are positively related to sourness (0.7362, *p* < 0.01). In addition, free amino acids have a significant impact on the instrumental taste profile of MRPs. TFAA is positively related to sourness (0.9060, *p* < 0.001) but negatively related to saltiness (−0.5917, *p* < 0.05), while the correlation with other types of free amino acids was not significant. The volatile components of esters showed significant correlations with the scores of various taste attributes in the instrumental taste profile, showing a positive correlation with umami (0.7293, *p* < 0.01) and sourness (0.6149, *p* < 0.05) and a negative correlation with saltiness (−0.7561, *p* < 0.01) and bitterness (−0.6972, *p* < 0.05). Furthermore, 2-isopropyl-5-methylhex-2-enal is positively related to bitterness (0.6535, *p* < 0.05) and saltiness (0.6503, *p* < 0.05), while pyrazine,2,5-dimethyl-3-(3-methylbutyl)- is negatively related to saltiness (−0.7382, *p* < 0.01) and bitterness (−0.5810, *p* < 0.05) but positively related to umami (0.6186, *p* < 0.05). The sweetness is positively related to hexanoic acid (0.6215, *p* < 0.05), p-cymen-7-ol (0.6141, *p* < 0.05), and 2,2′-Bifuran (0.5971, *p* < 0.05). Moreover, it should be noted that this experimental design did not include a control reaction containing only PPHs and xylose without added amino acids. Setting up this control could further distinguish the effects of MRPs derived from peptide-bound amino groups and those derived from added free amino acids. Future research should include a PPH + xylose control without added amino acid to further analyze the specific role of each ingredient of MR.

## 4. Conclusions

The EDU- and EGU-assisted enzymatic treatment of potato protein and different exogenous amino acids showed a significant influence on the MR progression, peptide structure, and antioxidant and instrumental taste profile. EDU and EGU promoted MR progression as indicated by decreased pH values, an increased browning degree, and enhanced fluorescence intensity. MRPs from EDU revealed significantly stronger umami and lower bitterness, while MRPs from EGU exhibited stronger sweetness. Under the equal-mass addition of exogenous amino acids, EDU-Ala-120 showed the highest levels of MR intermediates, most MW <2 kDa fractions, and the highest ORAC value among all MRPs. Therefore, the combination of EDU-assisted enzymatic treatment and MR with the addition of proper exogenous amino acid could be a tremendous technology to enhance the instrumental taste profile and antioxidant capacity of food protein. Furthermore, future research should include additional control systems to differentiate the roles of the peptide Maillard reaction, the xylose–amino acid reaction, and thermal degradation, thereby providing deeper mechanistic insights.

## Figures and Tables

**Table 1:** Changes in pH values and browning intensity of Maillard reaction products from potato protein hydrolysates as influenced by ultrasonic-assisted enzymatic treatment and exogenous amino acids.

Samples	pH	A_294_	A_420_
C-PPH	7.40 ± 0.02 ^a^	0.18 ± 0.00 ^f^	0.08 ± 0.00 ^h^
C-Ala-120	5.47 ± 0.02 ^c^	1.53 ± 0.14 ^de^	0.39 ± 0.01 ^g^
C-Phe-120	5.65 ± 0.03 ^b^	1.35 ± 0.03 ^e^	0.52 ± 0.01 ^ef^
C-Val-120	5.30 ± 0.02 ^e^	1.55 ± 0.02 ^de^	0.65 ± 0.01 ^c^
EDU-PPH	7.40 ± 0.01 ^a^	0.16 ± 0.00 ^f^	0.06 ± 0.00 ^i^
EDU-Ala-120	5.48 ± 0.02 ^c^	1.84 ± 0.05 ^bc^	0.54 ± 0.02 ^e^
EDU-Phe-120	5.34 ± 0.02 ^de^	1.84 ± 0.02 ^bc^	0.61 ± 0.01 ^d^
EDU-Val-120	5.40 ± 0.02 ^cd^	1.93 ± 0.04 ^bc^	0.63 ± 0.01 ^cd^
EGU-PPH	7.40 ± 0.02 ^a^	0.15 ± 0.00 ^f^	0.06 ± 0.00 ^hi^
EGU-Ala-120	5.41 ± 0.02 ^cd^	1.66 ± 0.03 ^cd^	0.51 ± 0.01 ^f^
EGU-Phe-120	5.38 ± 0.03 ^de^	2.12 ± 0.06 ^ab^	0.72 ± 0.02 ^b^
EGU-Val-120	5.34 ± 0.04 ^de^	2.31 ± 0.05 ^a^	0.82 ± 0.02 ^a^

Note: C, non-ultrasonicated conventional enzymatic hydrolysis; PPH, potato protein hydrolysates; EDU, energy-divergent ultrasound; EGU, energy-gathered ultrasound. Ala, Alanine; Phe, Phenylalanine; Val, Valine. Values followed by different superscript lowercase letters in the same column mean statistically significant differences (*p* < 0.05).

## Data Availability

The original contributions presented in this study are included in the article/Supplementary Materials. Further inquiries can be directed to the corresponding author.

## Supplementary Materials

The following supporting information can be downloaded at: https://www.mdpi.com/article/10.3390/foods15183300/s1, Table S1: Changes in pH values and browning intensity of Maillard reaction products from potato protein hydrolysates affected by ultrasound-assisted enzymatic treatment and exogenous amino acids. Table S2: Free amino acid content (FAA, g/100g) of non-ultrasonicated conventional (C), energy divergent ultrasound (EDU), and energy gathered ultrasound (EGU)-assisted enzymatic hydrolysis of potato protein hydrolysates (PPHs) and their Maillard reaction products. Table S3: Aroma compounds in non-ultrasonicated conventional (C), energy divergent ultrasound (EDU), and energy gathered ultrasound (EGU)-assisted enzymatic hydrolysis of potato protein hydrolysates (PPHs) and their Maillard reaction products (relative peak area, %). Figure S1: Pearson correlations between the molecular weight distribution, FAA contentsand aroma compounds (including individual volatile organic compounds) and flavor characteristics of PPHs and their MRPs. Table S4: Pearson correlations between the molecular weight distribution, FAAs contents, aroma compounds and flavor characteristics of PPHs and their MRPs.
