# Supplementary material for: Improvement of Ultrasound-Assisted Enzymatic Treatment and Exogenous Amino Acids on Structural, Antioxidant, and Instrumental Taste Profile of Maillard Reaction Products from Potato Protein Hydrolysates
Source: Foods. 2026 Sep 18;15(18):3300. doi: 10.3390/foods15183300 (PMC13606754; doi:10.3390/foods15183300)
Supplement: Supplementary file 1 [file foods-15-03300-s001.zip › foods-4499190-supplementary.pdf]

Supplementary material

Improvement of ultrasound-assisted enzymatic treatment and exogenous amino acids on structural, antioxidant and instrumental taste profile of Maillard reaction products from potato protein hydrolysates

Hui Liu [1†], Miao Zhang [1†], Taihua Mu [1, 2], Hongnan Sun [1*]

1 Laboratory of Food Chemistry and Nutrition Science, Institute of Food Science and Technology, Chinese Academy of Agricultural Sciences, Key Laboratory of Agro-Products Processing, Ministry of Agriculture and Rural Affairs, No. 2 Yuan Ming Yuan West Road, Haidian District, Beijing 100193, China.

2 College of Food Science and Technology, Yunnan Agricultural University, Kunming 650201, China.

[†] These authors contributed equally.

[*] Corresponding Author

Tel. & Fax: +(+86) 10 62815541.

E-Mail Address: sunhongnan@caas.cn (H.N. Sun); zhangmiao@caas.cn (M. Zhang); mutaihua@126.com (T.H. Mu).

Table S1 Changes in pH values and browning intensity of Maillard reaction products from potato protein hydrolysates affected by ultrasound-assisted enzymatic treatment and exogenous amino acids.

**Samples**	**pH**	**A_{294}**	**A_{420}**
C-PPH	7.40 ±0.02^{a}	0.18±0.00^{w}	0.08±0.00^{q}
C-Ala-30	6.63±0.01^{c}	0.50±0.01st	0.10±0.00^{pq}
C-Ala-60	6.23±0.02^{ef}	0.95±0.10^{op}	0.21±0.00^{m}
C-Ala-90	5.81±0.02^{i}	1.11±0.09^{l}	0.28±0.00^{k}
C-Ala-120	5.47±0.02^{n}	1.53±0.14^{f}	0.39±0.01^{h}
C-Phe-30	6.67±0.02^{bc}	0.46±0.01^{tu}	0.12±0.00^{o}
C-Phe-60	6.09±0.03^{g}	1.00±0.05^{no}	0.24±0.01^{l}
C-Phe-90	5.85±0.04^{hi}	1.08±0.02^{lm}	0.35±0.00^{i}
C-Phe-120	5.65±0.03^{jkl}	1.35±0.03^{hi}	0.52±0.01^{f}
C-Val-30	6.70±0.02^{bc}	0.46±0.02^{tu}	0.19±0.00^{n}
C-Val-60	6.09±0.04^{g}	0.74±0.03^{q}	0.21±0.01^{m}
C-Val-90	5.63±0.03^{kl}	1.26±0.09^{jk}	0.31±0.01^{j}
C-Val-120	5.30±0.02^{o}	1.55±0.02^{f}	0.65±0.01^{c}
EDU-PPH	7.40±0.01^{a}	0.16±0.00^{w}	0.06±0.00^{r}
EDU-Ala-30	6.66±0.04^{c}	0.43±0.00^{uv}	0.09±0.00^{q}
EDU-Ala-60	6.14±0.04^{fg}	0.93±0.00^{p}	0.21±0.00^{m}
EDU-Ala-90	5.85±0.02^{hi}	1.38±0.01^{gh}	0.35±0.00^{i}
EDU-Ala-120	5.48±0.02^{mn}	1.84±0.05^{d}	0.54±0.02^{e}
EDU-Phe-30	6.44±0.02^{d}	0.49±0.01^{stu}	0.11±0.00^{op}
EDU-Phe-60	5.92±0.01^{h}	1.01±0.00^{mno}	0.25±0.00^{l}
EDU-Phe-90	5.72±0.03^{j}	1.62±0.02^{e}	0.52±0.01^{f}
EDU-Phe-120	5.34±0.02^{no}	1.84±0.02^{d}	0.61±0.01^{d}
EDU-Val-30	6.67±0.01^{bc}	0.54±0.01^{rs}	0.11±0.00^{op}
EDU-Val-60	6.09±0.05^{g}	1.21±0.01^{k}	0.30±0.00^{j}
EDU-Val-90	5.71±0.01^{jk}	1.44±0.01^{g}	0.42±0.00^{g}
EDU-Val-120	5.40±0.02^{mn}	1.93±0.04^{c}	0.63±0.01^{d}
EGU-PPH	7.40±0.02^{a}	0.15±0.00^{w}	0.06±0.00^{r}
EGU-Ala-30	6.76±0.00^{b}	0.38±0.02^{v}	0.11±0.00^{op}
EGU-Ala-60	6.25±0.04^{e}	0.79±0.02^{q}	0.20±0.00^{m}
EGU-Ala-90	5.88±0.03^{hi}	1.31±0.04^{ij}	0.41±0.01^{g}
EGU-Ala-120	5.41±0.02^{mn}	1.66±0.03^{e}	0.51±0.01^{f}

EGU-Phe-30	6.68±0.02^{bc}	0.46±0.01^{tu}	0.13±0.01^{o}
EGU-Phe-60	6.20±0.02^{ef}	1.00±0.02^{no}	0.27±0.01^{k}
EGU-Phe-90	5.59±0.03^{l}	1.39±0.05^{gh}	0.42±0.02^{g}
EGU-Phe-120	5.38±0.03^{no}	2.12±0.06^{b}	0.72±0.02^{b}
EGU-Val-30	6.65±0.03^{c}	0.61±0.03^{r}	0.21±0.00^{m}
EGU-Val-60	6.07±0.05^{g}	1.05±0.04^{lmn}	0.35±0.01^{i}
EGU-Val-90	5.66±0.06^{jkl}	1.26±0.02^{jk}	0.42±0.00^{g}
EGU-Val-120	5.34±0.04^{no}	2.31±0.05^{a}	0.82±0.02^{a}

C, non-ultrasonicated conventional enzymatic hydrolysis; EDU, energy divergent ultrasound; EGU, energy gather ultrasound. Ala, Alanine; Phe, Phenylalanine; Val, Valine. 30/60/90/120, Maillard reaction time 30, 60, 90 and 120 min. Values followed by different superscript lowercase letters in the same column mean statistically significant differences (P < 0.05).

Table S2. Free amino acid content (FAA, g/100g) of non-ultrasonicated conventional (C), energy divergent ultrasound (EDU), and energy gathered ultrasound (EGU)-assisted enzymatic hydrolysis of potato protein hydrolysates (PPHs) and their Maillard reaction products

Amino acids	C-PPH	C-Ala-120	C-Phe-120	C-Val-120	EDU-PPH	EDU-Ala-120	EDU-Phe-120	EDU-Val-120	EGU-PPH	EGU-Ala-120	EGU-Phe-120	EGU-Val-120
Asp	0.09±0.00^{fg}	0.13±0.00^{bcd}	0.12±0.00^{d}	0.13±0.00^{b}	0.09±0.00^{g}	0.13±0.00^{bc}	0.11±0.00^{e}	0.13±0.00^{bcd}	0.09±0.00^{f}	0.15±0.00^{a}	0.12±0.00^{cd}	0.14±0.00^{a}
Thr	0.55±0.00^{b}	0.37±0.00^{f}	0.36±0.00^{g}	0.36±0.00^{h}	0.53±0.00^{c}	0.33±0.00^{i}	0.30±0.00^{k}	0.31±0.00^{j}	0.57±0.00^{a}	0.42±0.00^{d}	0.37±0.00^{f}	0.42±0.00^{e}
Ser	0.17±0.00^{f}	0.17±0.00^{g}	0.16±0.00^{gh}	0.16±0.00^{h}	0.18±0.00^{e}	0.17±0.00^{g}	0.15±0.00^{i}	0.16±0.00^{h}	0.32±0.00^{a}	0.23±0.00^{b}	0.20±0.00^{d}	0.22±0.00^{c}
Glu	0.38±0.00^{c}	0.35±0.00^{d}	0.35±0.00^{d}	0.34±0.00^{e}	0.38±0.00^{c}	0.36±0.00^{d}	0.38±0.00^{c}	0.34±0.00^{e}	0.43±0.00^{a}	0.41±0.00^{b}	0.42±0.00^{ab}	0.41±0.00^{b}
Pro	0.01±0.00^{h}	0.01±0.00^{g}	0.01±0.00^{f}	0.02±0.00^{a}	0.01±0.00^{g}	0.01±0.00^{bc}	0.01±0.00^{ef}	0.01±0.00^{ef}	0.01±0.00^{i}	0.01±0.00^{cd}	0.02±0.00^{ab}	0.01±0.00^{de}
Gly	0.06±0.00^{c}	-	0.04±0.00^{h}	0.04±0.00^{g}	0.07±0.00^{b}	-	0.04±0.00^{h}	0.04±0.00^{f}	0.10±0.00^{a}	-	0.05±0.00^{e}	0.06±0.00^{d}
Ala	0.27±0.01^{de}	10.76±0.04^{a}	0.18±0.00^{e}	0.19±0.00^{e}	0.27±0.00^{de}	10.54±0.13^{b}	0.17±0.00^{e}	0.19±0.00^{e}	0.35±0.00^{d}	10.36±0.04^{c}	0.21±0.00^{e}	0.23±0.00^{de}
Val	0.59±0.01^{e}	0.39±0.01^{hi}	0.37±0.01^{i}	10.08±0.02^{b}	0.54±0.01^{ef}	0.37±0.01^{i}	0.34±0.01^{i}	9.90±0.01^{c}	0.66±0.00^{d}	0.50±0.01^{fg}	0.45±0.02^{gh}	10.16±0.01^{a}
Met	0.24±0.01^{b}	0.12±0.01^{f}	0.12±0.01^{ef}	0.12±0.02^{ef}	0.21±0.01^{c}	0.11±0.01^{f}	0.09±0.01^{g}	0.12±0.01^{ef}	0.29±0.00^{a}	0.16±0.01^{d}	0.13±0.02^{e}	0.17±0.01^{d}
Ile	0.39±0.00^{b}	0.26±0.00^{ef}	0.25±0.00^{fg}	0.26±0.00^{ef}	0.39±0.00^{b}	0.25±0.00^{g}	0.23±0.00^{h}	0.25±0.00^{g}	0.43±0.00^{a}	0.34±0.00^{c}	0.31±0.00^{d}	0.34±0.00^{c}
Leu	1.23±0.01^{d}	0.85±0.01^{h}	0.84±0.01^{h}	0.96±0.01^{g}	1.30±0.01^{b}	0.85±0.01^{h}	0.80±0.01^{i}	0.96±0.01^{g}	1.31±0.00^{a}	1.13±0.01^{e}	1.05±0.01^{f}	1.27±0.01^{c}
Tyr	0.80±0.00^{b}	0.53±0.00^{e}	0.49±0.00^{fg}	0.48±0.00^{g}	0.85±0.00^{a}	0.50±0.00^{f}	0.45±0.00^{h}	0.45±0.00^{h}	0.85±0.00^{a}	0.62±0.00^{c}	0.55±0.00^{d}	0.62±0.00^{c}
Phe	1.89±0.01^{e}	1.13±0.01^{h}	10.49±0.01^{a}	1.02±0.01^{i}	2.03±0.01^{d}	1.09±0.01^{h}	9.83±0.01^{b}	1.00±0.01^{i}	1.45±0.00^{f}	1.22±0.01^{g}	7.51±0.01^{c}	1.22±0.01^{g}
Lys	0.61±0.00^{b}	0.36±0.00^{e}	0.32±0.00^{h}	0.31±0.00^{i}	0.61±0.00^{b}	0.33±0.00^{g}	0.27±0.00^{k}	0.29±0.00^{j}	0.69±0.00^{a}	0.40±0.00^{c}	0.34±0.00^{f}	0.38±0.00^{d}
His	0.24±0.00^{a}	0.12±0.00^{d}	0.12±0.00^{e}	0.12±0.00^{f}	0.21±0.00^{b}	0.11±0.00^{f}	0.09±0.00^{g}	0.12±0.00^{g}	0.21±0.00^{c}	0.16±0.00^{e}	0.13±0.00^{f}	0.17±0.00^{e}
Arg	0.80±0.00^{b}	0.32±0.00^{d}	0.29±0.00^{ef}	0.26±0.00^{g}	0.84±0.00^{a}	0.28±0.00^{f}	0.23±0.00^{h}	0.24±0.00^{h}	0.69±0.00^{c}	0.33±0.00^{d}	0.30±0.00^{e}	0.32±0.00^{d}
UFAA	0.80±0.01^{de}	11.24±0.04^{a}	0.69±0.00^{e}	0.69±0.01^{e}	0.81±0.00^{de}	11.02±0.14^{b}	0.70±0.00^{e}	0.70±0.01^{e}	0.97±0.00^{c}	10.91±0.04^{b}	0.80±0.00^{de}	0.85±0.00^{cd}
SFAA	1.06±0.01^{d}	11.30±0.05^{a}	0.74±0.00^{fg}	0.74±0.00^{fg}	1.06±0.00^{d}	11.03±0.13^{b}	0.65±0.00^{g}	0.71±0.00^{fg}	1.33±0.01^{c}	11.01±0.04^{b}	0.83±0.00^{ef}	0.93±0.00^{de}
BFAA	5.14±0.02^{g}	3.28±0.03^{j}	12.56±0.06^{c}	12.93±0.09^{b}	5.33±0.01^{f}	3.18±0.03^{j}	11.74±0.07^{d}	12.67±0.04^{c}	4.99±0.01^{h}	3.97±0.02^{i}	10.01±0.03^{e}	13.77±0.03^{a}
TFAA	8.33±0.03^{h}	15.88±0.08^{b}	14.51±0.07^{e}	14.82±0.09^{d}	8.55±0.00^{h}	15.42±0.17^{c}	13.48±0.07^{f}	14.47±0.04^{e}	8.43±0.03^{h}	16.39±0.03^{a}	12.13±0.03^{g}	16.07±0.04^{b}

Note: Ala, Alanine; Phe, Phenylalanine; Val, Valine. 120, Maillard reaction time 120 min. TFAA, total free amino acid content; SFAA, Sweet free amino acid content; BFAA, Bitter free amino acid content; UFAA, Umami free amino acid content. Values followed by different superscript lowercase letters in the same column mean statistically significant differences ($p < 0.05$).

Table S3. Aroma compounds in non-ultrasonicated conventional (C), energy divergent ultrasound (EDU), and energy gathered ultrasound (EGU)-assisted enzymatic hydrolysis of potato protein hydrolysates (PPHs) and their Maillard reaction products (relative peak area, %)

Code	RT	Names	C-PPH	C-Ala-120	C-Phe-120	C-Val-120	EDU-PPH	EDU-Ala-120	EDU-Phe-120	EDU-Val-120	EGU-PPH	EGU-Ala-120	EGU-Phe-120	EGU-Val-120
		**Aldehydes**	**14.27±1.00^{f}**	**40.11±0.60^{de}**	**58.59±0.61^{b}**	**42.89±0.82^{cd}**	**9.24±0.11^{g}**	**43.09±1.92^{cd}**	**58.79±0.34^{b}**	**36.8±1.37^{e}**	**12.02±0.69^{fg}**	**55.22±0.14^{b}**	**65.80±1.08^{a}**	**46.15±2.90^{c}**
F1	3.54	Butanal, 3-methyl-	-	-	-	1.26±0.02^{ab}	-	-	-	1.35±0.12^{a}	-	0.40±0.01^{d}	0.61±0.04^{c}	1.15±0.11^{b}
F2	6.17	Hexanal	2.70±0.06^{a}	1.43±0.00^{d}	0.55±0.00^{hi}	0.90±0.07^{fg}	1.87±0.03^{c}	1.38±0.11^{de}	0.49±0.04^{i}	0.97±0.09^{f}	2.14±0.15^{b}	1.19±0.01^{e}	0.40±0.02^{i}	0.72±0.06^{gh}
F3	13.64	2-Isopropyl-5-methylhex-2-enal	-	-	0.10±0.00b	-	-	-	-	0.54±0.00^{a}	-	-	-	0.10±0.01^{b}
F4	14.57	Nonanal	-	8.88±1.26^{a}	1.96±0.06ef	4.54±0.18^{c}	-	4.86±0.50^{c}	1.84±0.04^{eg}	3.16±0.07^{de}	2.37±0.30^{ef}	6.80±0.01^{b}	1.74±0.08^{f}	4.19±0.31^{cd}
F5	15.51	2-Octenal, (E)-	3.36±0.09^{a}	0.37±0.02^{def}	0.14±0.01fg	0.25±0.01^{defg}	1.79±0.07^{c}	0.52±0.02^{d}	-	0.17±0.01^{efg}	2.80±0.31^{b}	0.47±0.01^{de}	-	0.19±0.02^{efg}
F6	16.32	Furfural	-	5.28±0.20^{de}	6.73±0.76^{cde}	9.18±1.14^{bc}	-	6.19±0.37^{de}	4.46±0.09^{e}	13.67±0.59^{a}	-	7.73±0.01^{cd}	10.62±0.37^{b}	13.60±2.38^{a}
F7	16.61	3-Cyclohexene-1-carboxaldehyde, 1-methyl-	-	0.16±0.01^{b}	-	-	-	0.18±0.00^{b}	-	-	-	0.25±0.07^{a}	-	-
F8	17.36	Decanal	-	8.26±0.10^{b}	3.96±0.06^{e}	6.67±0.20^{c}	-	9.89±0.80^{a}	5.35±0.13^{d}	-	-	8.66±0.04^{b}	3.47±0.20^{e}	5.84±0.46^{cd}
F9	17.78	Benzaldehyde	8.21±0.97^{de}	10.78±1.83^{cd}	15.88±1.42^{a}	10.03±1.88^{d}	5.58±0.01^{ef}	14.45±0.87^{ab}	17.04±0.36^{a}	11.43±0.39^{bcd}	4.72±0.07^{f}	13.93±0.00^{abc}	15.52±0.46^{a}	9.40±0.74^{d}
F10	19.97	1H-Pyrazole-4-carboxaldehyde,1,5-dimethyl-	-	1.45±0.05^{b}	0.70±0.01^{d}	1.20±0.00^{c}	-	1.67±0.12^{a}	0.78±0.08^{d}	-	-	1.05±0.00^{c}	0.51±0.03^{e}	1.22±0.13^{c}
F11	20.05	U-ecanal	-	1.19±0.08^{a}	0.36±0.00^{e}	0.94±0.06^{b}	-	0.48±0.01^{d}	0.26±0.03^{ef}	-	-	0.74±0.00^{c}	0.16±0.01^{f}	0.54±0.06^{e}
F12	20.70	Benzeneacetaldehyde	-	1.21±0.12^{cd}	11.37±0.75^{b}	1.16±0.02^{cd}	-	1.45±0.06^{c}	13.50±0.68^{a}	1.46±0.05^{c}	-	0.99±0.02^{cd}	13.67±0.99^{a}	1.32±0.12^{cd}
F13	21.54	2-Octenal, 2-butyl-	-	-	-	0.73±0.02^{a}	-	-	0.67±0.01^{b}	-	-	-	-	-
F14	22.58	Heptadecanal	-	-	-	0.70±0.06^{a}	-	-	-	-	-	-	-	-
F15	23.92	Benzaldehyde,4-(1-methylethyl)-	-	-	4.52±0.14^{c}	3.82±0.29^{d}	-	-	-	-	-	11.54±0.02^{a}	5.34±0.04^{b}	-
F16	23.95	Propanal, 2-methyl-3-phenyl-	-	-	-	-	-	-	-	-	-	-	-	6.14±0.99^{a}
F17	24.55	Benzaldehyde,2,4-dimethyl-	-	-	-	-	-	0.26±0.03^{b}	3.52±0.15^{a}	0.14±0.00^{bc}	-	0.21±0.00^{b}	-	-
F18	25.93	2-Methyl-3(2-furyl)acrolein	-	-	-	-	-	-	-	-	-	-	-	0.20±0.02^{a}
F19	26.68	(2-Methyl-cyclohex-2-enylidene)-acetaldehyde	-	-	-	-	-	0.30±0.01^{a}	-	-	-	0.27±0.00^{b}	-	-
F20	27.08	Benzeneacetaldehyde,.alpha.-ethylidene-	-	1.11±0.03^{c}	2.70±0.07^{a}	0.13±0.00^{d}	-	0.92±0.33^{c}	2.33±0.02^{b}	0.24±0.00^{d}	-	0.99±0.01^{c}	2.97±0.17^{a}	0.13±0.02^{d}
F21	27.35	Benzeneacetaldehyde,.alpha.-(2-methylpropylidene)-	-	-	0.82±0.06^{c}	0.89±0.02^{c}	-	-	1.01±0.07^{b}	1.53±0.08^{a}	-	-	0.91±0.05^{bc}	0.89±0.07^{bc}

F22	27.81	trans-U-ec-4-enal	-	-	-	-	-	-	-	0.24±0.01^{a}	-	-	-	-
F23	31.08	5-Methyl-2-phenyl-2-hexenal	-	-	8.34±1.07^{ab}	0.49±0.01^{cd}	-	0.54±0.09^{cd}	7.13±0.81^{b}	1.90±0.79^{c}	-	-	8.99±0.60^{a}	0.51±0.01^{cd}
F24	39.76	2-Phenyl-3-(2-furyl)-propenal	-	-	0.46±0.00^{b}	-	-	-	0.44±0.11^{b}	-	-	-	0.89±0.04^{a}	-
		**Ketones**	**25.23±1.47^{a}**	**16.66±0.17^{cd}**	**13.59±0.19^{efg}**	**15.53±0.13^{def}**	**25.51±0.28^{a}**	**19.02±0.14^{bc}**	**12.94±0.37^{fg}**	**16.53±0.37^{cd}**	**21.21±1.15^{b}**	**14.30±0.04^{def}**	**11.12±0.32^{fg}**	**13.92±2.28^{defg}**
F25	8.76	2-Heptanone	9.65±1.45^{b}	4.80±0.14^{d}	2.27±0.05^{ef}	4.05±0.14^{de}	12.95±0.36^{a}	5.35±0.10^{cd}	2.09±0.07^{f}	1.86±0.07^{f}	6.65±0.37^{c}	4.47±0.01^{d}	1.58±0.07^{f}	2.72±0.18^{f}
F26	10.54	3-Hexen-2-one, 5-methyl-	-	-	-	0.20±0.01^{b}	-	-	-	0.45±0.01^{a}	-	-	-	-
F27	11.45	2-Butanone,1-(2-furanyl)-3-methyl-	-	-	-	0.17±0.01^{a}	-	-	-	-	-	-	-	-
F28	11.47	2,3-Octanedione	-	0.19±0.01^{c}	-	-	-	0.34±0.03^{b}	0.33±0.00^{b}	-	1.18±0.11^{a}	-	-	0.14±0.02^{c}
F29	11.63	2-Octanone	-	1.17±0.00^{c}	0.71±0.06^{d}	1.07±0.01^{c}	2.70±0.04^{a}	1.23±0.06^{c}	0.43±0.01^{e}	0.72±0.03^{d}	1.81±0.17^{b}	1.08±0.01^{c}	0.40±0.02^{e}	0.74±0.04^{d}
F30	12.19	2-Propanone,1-hydroxy-	-	0.32±0.01^{d}	0.20±0.01^{e}	0.49±0.02^{c}	-	0.57±0.06^{b}	0.23±0.01^{e}	0.78±0.03^{a}	-	0.13±0.00^{f}	0.08±0.00^{f}	0.31±0.00^{d}
F31	12.69	3-Octanone,2-methyl-	-	-	-	-	-	-	0.20±0.01^{a}	-	-	-	-	-
F32	12.71	2,5-Hexanedione	-	-	-	-	-	-	-	-	-	0.21±0.01^{a}	-	-
F33	12.72	2,5-Octanedione	-	0.27±0.01^{a}	-	-	-	-	-	-	-	-	0.07±0.00^{b}	-
F34	13.03	5-Hepten-2-one, 6-methyl-	-	-	0.04±0.00^{a}	-	-	-	-	-	-	-	-	-
F35	14.51	2-Nonanone	2.91±0.11^{a}	-	-	0.08±0.00^{c}	0.72±0.01^{b}	-	-	-	-	-	-	0.06±0.01^{c}
F36	14.96	3-Octen-2-one	0.59±0.04^{a}	-	-	-	-	-	0.16±0.02^{b}	-	-	-	-	-
F37	16.89	2-Nonen-4-one	-	1.05±0.03^{a}	0.65±0.00^{c}	0.79±0.03^{b}	-	0.28±0.01^{e}	0.19±0.00^{f}	0.19±0.00^{f}	-	0.63±0.00^{c}	0.29±0.02^{e}	0.42±0.03^{d}
F38	16.97	3-Heptanone, 2-methyl-	-	-	-	-	-	-	-	0.12±0.00^{a}	-	-	-	-
F39	17.37	Ethanone, 1-(2-furanyl)-	-	-	-	-	-	-	-	1.03±0.06^{a}	-	-	-	-
F40	17.69	trans-3-Nonen-2-one	0.93±0.15^{de}	1.86±0.11^{a}	1.05±0.00^{cd}	-	1.16±0.01^{bc}	1.71±0.00^{a}	0.81±0.00^{e}	1.16±0.06^{bc}	0.97±0.11^{cde}	1.35±0.01^{b}	-	-
F41	18.10	2-Butanone, 3,3-dimethyl-	-	-	-	-	-	0.27±0.02^{a}	-	-	-	-	-	-
F42	19.05	3,5-Octadien-2-one, (E,E)-	2.91±0.17^{a}	0.39±0.03^{cd}	0.20±0.01^{de}	-	2.24±0.01^{b}	-	0.27±0.03^{de}	0.64±0.03^{c}	2.430.32^{b}	-	-	-
F43	19.15	Ethanone, 1-(3-methylenecyclopentyl)-	-	-	-	-	-	0.41±0.04^{a}	-	-	-	0.31±0.00^{b}	-	0.19±0.01^{c}
F44	19.20	1-Propanone, 1-(2-furanyl)-	-	0.54±0.03^{a}	0.15±0.01d	0.41±0.01^{b}	-	0.41±0.04^{b}	0.15±0.02^{d}	0.38±0.01^{b}	-	0.25±0.00^{c}	0.25±0.00^{c}	0.17±0.01^{d}
F45	20.92	Acetophenone	-	-	2.38±0.18^{b}	-	-	-	2.81±0.07^{a}	-	-	-	2.61±0.13^{a}	-
F46	21.73	2-methyl-Cyclopentadecanone	-	-	-	-	-	-	-	0.53±0.03^{a}	-	-	-	-

F47	22.69	Benzyl methyl ketone	-	-	0.22±0.01b	-	-	-	0.28±0.01^{a}	-	-	-	0.13±0.01^{c}	-
F48	23.14	2(5H)-Furanone	-	0.43±0.01^{b}	0.22±0.00^{c}	0.42±0.02^{b}	-	-	0.40±0.01^{b}	0.38±0.01^{b}	-	-	-	0.53±0.08^{a}
F49	23.51	2(3H)-Furanone,5-dihydro-5-methyl-	-	-	-	-	-	-	0.11±0.01^{a}	-	-	-	-	-
F50	23.77	Nona-3,5-dien-2-one	-	-	-	1.00±0.05^{b}	-	-	-	-	-	-	-	1.41±0.30^{a}
F51	24.17	Isophorone	-	-	-	-	-	-	0.13±0.01^{b}	0.68±0.02^{a}	-	-	-	-
F52	24.52	1,2-Propanedione, 1-phenyl	-	-	1.43±0.03^{a}	-	-	-	-	-	-	-	1.00±0.04^{b}	-
F53	24.69	Tetrahydrocarvone	-	0.15±0.00^{b}	0.20±0.01^{a}	-	-	-	-	-	-	-	-	-
F54	24.71	3-Ethoxy-2-methyl-2-cyclohexen-1-one	-	-	-	0.16±0.02^{a}	-	-	-	-	-	-	-	-
F55	24.71	2-Sec-Butylcyclohexanone	-	-	-	-	-	0.39±0.30^{a}	-	-	-	-	-	-
F56	24.72	Cyclohexanone, 2-octyl	-	-	-	-	-	-	0.10±0.01^{b}	-	-	0.15±0.00^{a}	-	-
F57	25.68	(Z)-6,10-dimethyl-5,9-U-ecadien-2-one	2.70±0.38^{a}	-	-	-	1.04±0.01^{b}	-	-	-	2.80±0.35^{a}	-	-	-
F58	25.69	5,9-U-ecadien-2-one,6,10-dimethyl-	-	0.42±0.02^{b}	0.95±0.01^{ab}	0.27±0.02^{b}	-	0.72±0.03^{ab}	0.86±0.06^{ab}	0.43±0.02^{b}	-	0.62±0.00^{ab}	0.86±0.05^{ab}	3.01±2.48^{a}
F59	26.53	3-Buten-2-one,4-(2-furanyl)-	-	1.09±0.02^{c}	1.00±0.01^{cde}	1.29±0.03^{b}	-	1.05±0.02^{cd}	0.85±0.06^{de}	2.42±0.17^{a}	-	0.81±0.00^{e}	1.03±0.06^{cd}	1.47±0.12^{b}
F60	26.68	Bicyclo[3.3.0]octan-2-one, 7-isopropylidene	-	-	-	-	-	-	0.37±0.03^{a}	-	-	-	-	-
F61	26.70	Bicyclo[3.1.0]hex-3-en-2-one	-	0.42±0.01^{a}	-	-	-	-	-	-	-	-	-	-
F62	26.70	2(1H)-Azulenone,4,5,6,7,8,8a-hexahydro-8a-methyl-	-	-	0.46±0.00^{c}	0.52±0.03^{c}	-	-	-	0.91±0.01^{a}	-	-	0.46±0.02^{c}	0.63±0.05^{b}
F63	29.16	2-Pentadecanone	-	0.23±0.05^{a}	-	0.12±0.01^{b}	-	-	-	-	-	-	-	-
F64	29.17	2-Tridecanone	-	-	-	-	-	-	0.13±0.01^{c}	-	-	0.18±0.00^{a}	0.16±0.00^{b}	-
F65	30.63	1,3-Dioxol-2-one,4,5-dimethyl-	-	1.79±0.05^{e}	0.82±0.01^{g}	3.52±0.11^{b}	-	4.79±0.21^{a}	1.00±0.01^{g}	2.41±0.00^{d}	-	2.70±0.02^{c}	1.65±0.13^{e}	1.31±0.05^{f}
F66	37.77	Solavetivone	5.53±0.07^{a}	1.55±0.01^{c}	0.64±0.04^{d}	0.99±0.03^{cd}	4.70±0.02^{b}	1.50±0.06^{c}	1.05±0.01^{cd}	1.44±0.06^{c}	5.37±0.58^{a}	1.40±0.02^{c}	0.56±0.02^{d}	0.83±0.04^{d}
		**Esters**	**-**	**10.61±0.92a**	**0.21±0.01^{e}**	**0.35±0.00^{e}**	**-**	**7.09±1.74^{b}**	**1.33±0.14^{de}**	**9.00±0.31^{ab}**	**2.12±0.25^{cde}**	**4.14±0.01^{c}**	**0.89±0.04^{de}**	**2.48±0.89^{cd}**
F67	13.06	heptafluorobutyrate	-	0.59±0.00^{a}	-	-	-	-	-	-	-	-	-	-
F68	20.82	Menthyl acetate	-	-	-	-	-	-	-	-	-	2.36±0.01^{a}	-	-
F69	20.95	Pentafluoropropionic acid, hexadecyl ester	-	-	-	-	-	-	-	-	-	0.65±0.02^{a}	-	-
F70	21.47	6-Bromohexanoic acid,heptyl ester	-	-	-	-	-	-	-	-	-	-	-	1.61±0.23^{a}
F71	23.77	3-Hydroxypyridinemonoacetate	-	-	-	-	-	-	-	-	-	-	0.76±0.04^{a}	-

F72	23.87	4-Ethylbenzoic acid,cyclopentyl ester	-	9.81±0.92^{a}	-	-	-	7.09±1.74^{b}	-	8.89±0.32^{a}	-	-	-	-
F73	28.23	2-Nitrophenyl-.beta.-phenylpropionate	-	-	-	-	-	-	0.18±0.01^{a}	-	-	-	-	-
F74	33.36	Isopropyl palmitate	-	-	-	-	-	-	0.57±0.06^{a}	-	-	-	-	-
F75	33.62	Hexadecanoic acid,ethyl ester	-	-	-	-	-	-	0.40±0.08^{c}	-	1.45±0.11^{a}	0.70±0.03^{b}	0.07±0.00^{d}	-
F76	36.86	9-Octadecenoic acid,methyl ester, (E)-	-	-	-	-	-	-	-	-	-	0.22±0.01^{a}	-	-
F77	36.86	11-Octadecenoic acid,methyl ester	-	-	-	-	-	-	0.10±0.00^{a}	-	-	-	-	-
F78	38.23	1,2-Benzenedicarboxylic (2-methylpropyl) ester	-	0.21±0.01^{abc}	0.13±0.00^{bc}	0.26±0.00^{abc}	-	-	-	-	0.68±0.14^{ab}	0.22±0.01^{abc}	0.06±0.00^{bc}	0.79±0.65^{a}
F79	40.68	Dibutyl phthalate	-	-	0.08±0.00^{c}	0.09±0.01^{b}	-	-	0.09±0.01^{b}	0.11±0.00^{a}	-	-	-	0.08±0.00^{c}
		**Alcohols**	**4.80±0.08^{b}**	**3.29±0.05^{c}**	**6.68±0.00^{a}**	**2.54±0.08^{b}**	**3.17±0.00^{b}**	**4.37±0.05^{b}**	**6.61±0.29^{a}**	**6.53±0.38^{a}**	**2.84±0.09c**	**1.75±0.03^{d}**	**6.57±0.28^{a}**	**7.01±0.57^{a}**
F80	11.82	2-Hexanol, 3,4-dimethyl-	-	-	-	-	-	0.30±0.01^{a}	-	-	-	-	-	-
F81	13.74	1-Hexanol	0.98±0.04a	-	-	-	-	-	-	-	-	-	-	-
F82	14.97	3,5-Octadien-2-ol	-	0.69±0.02^{a}	-	-	0.48±0.03^{b}	-	-	0.42±0.01^{bc}	-	0.71±0.01^{a}	0.22±0.01^{d}	0.41±0.05^{c}
F83	16.22	1-Octen-3-ol	2.89±0.07^{a}	-	-	-	2.69±0.03^{a}	-	-	-	1.35±0.16^{b}	-	-	-
F84	18.69	1,6-Octadien-3-ol,3,7-dimethyl-	-	0.23±0.01^{c}	0.13±0.00^{e}	0.12±0.01^{e}	-	0.20±0.03^{cd}	0.17±0.02^{d}	0.32±0.01^{a}	-	0.27±0.01^{b}	0.12±0.01^{e}	0.19±0.01^{d}
F85	18.98	1-Octanol	0.63±0.08^{a}	0.42±0.02^{b}	0.20±0.00^{ef}	-	-	0.26±0.03^{de}	0.17±0.02^{f}	0.38±0.01^{bc}	-	0.33±0.00^{cd}	0.12±0.00^{f}	0.27±0.02^{de}
F86	19.03	1-Hexanol, 3-methyl-	-	-	-	0.25±0.01^{a}	-	-	-	-	-	-	-	-
F87	19.90	Orcinol	-	-	-	-	-	-	-	2.38±0.17^{a}	-	-	-	-
F88	20.35	3-Nonen-1-ol, (Z)-	-	-	-	0.25±0.01^{a}	-	-	-	-	-	-	-	-
F89	20.85	Cyclohexanol, 5-methyl-2-(1-methylethyl)-, acetate,	-	-	3.42±0.07^{ab}	-	-	-	3.31±0.18^{b}	-	-	-	3.67±0.18^{ab}	3.85±0.45^{a}
F90	21.25	2-Furanmethanol	-	1.95±0.04^{c}	1.33±0.05^{d}	1.92±0.08^{c}	-	2.33±0.05b	1.85±0.02^{c}	2.74±0.18^{a}	-	-	1.36±0.06^{cd}	1.82±0.16^{c}
F91	21.44	E-11,13-Tetradecadien-1-ol	-	-	0.46±0.04^{a}	-	-	-	-	-	-	-	-	-
F92	21.44	1-Nonanol	-	-	-	-	-	0.84±0.03^{a}	-	-	0.74±0.18^{b}	-	-	-
F93	21.87	2,4-Pentadien-1-ol,3-pentyl-, (2Z)-	-	-	-	-	-	-	-	-	-	-	-	0.35±0.04^{a}
F94	25.97	Benzyl alcohol	0.30±0.03^{d}	-	0.86±0.00^{a}	-	-	0.44±0.01^{c}	0.92±0.08^{a}	0.28±0.01^{d}	-	-	0.64±0.04^{b}	-
F95	28.09	1-Dodecanol	-	-	0.06±0.00^{a}	-	-	-	-	-	-	-	0.03±0.00^{b}	-
F96	29.56	Cyclopropanol, 2,2-dimethyl-3-(2-phenylethynyl)-	-	-	0.08±0.00^{b}	-	-	-	0.09±0.00^{b}	-	-	-	0.12±0.00^{a}	-

F97	30.50	p-Cymen-7-ol	-	-	-	-	-	-	-	-	-	-	$0.12±0.00^{a}$	$0.11±0.00^{a}$
F98	33.23	2-Phenyl-2,4-octadienol	-	-	$0.14±0.01^{a}$	-	-	-	$0.10±0.01^{b}$	-	-	-	$0.13±0.00^{a}$	-
F99	35.73	1-Hexadecanol	-	-	-	-	-	-	-	-	$0.76±0.11^{a}$	$0.44±0.00^{b}$	$0.04±0.00^{c}$	-
		**Acids**	**$5.76±0.49^{b}$**	**$2.37±0.09^{f}$**	**$2.21±0.00^{f}$**	**$4.70±0.27^{c}$**	**$3.73±0.01^{cde}$**	**$3.57±0.01^{de}$**	**$3.10±0.15^{ef}$**	**$6.17±0.20^{b}$**	**$16.45±0.90^{a}$**	**$3.03±0.01^{ef}$**	**$4.33±0.17^{cd}$**	**$4.02±0.16^{cde}$**
F100	16.13	Acetic acid	-	-	-	-	-	-	$1.67±0.08^{b}$	$2.57±0.15^{a}$	-	-	$1.18±0.06^{c}$	-
F101	25.34	Hexanoic acid	-	$0.30±0.01^{b}$	-	$0.27±0.02^{b}$	-	$0.27±0.02^{b}$	-	-	-	$0.32±0.00^{b}$	$0.24±0.00^{b}$	$0.40±0.09^{a}$
F102	26.19	Propanoic acid,2-methyl-	$5.76±0.49^{b}$	-	$0.88±0.00^{def}$	$0.50±0.02^{ef}$	$3.73±0.01^{cde}$	$1.80±0.09^{d}$	$0.43±0.04^{ef}$	$1.07±0.06^{de}$	$16.45±0.90^{a}$	$1.07±0.01^{de}$	$0.96±0.05^{def}$	$1.08±0.09^{de}$
F103	29.73	Octanoic acid	-	$1.68±0.07^{c}$	$1.15±0.00^{de}$	$1.58±0.11^{c}$	-	$1.51±0.08^{c}$	$0.99±0.02^{e}$	$2.52±0.01^{a}$	-	$1.64±0.01^{c}$	$1.30±0.06^{d}$	$1.99±0.13^{b}$
F104	31.79	Nonanoic acid	-	$0.39±0.01^{c}$	$0.18±0.01^{e}$	$1.05±0.02^{a}$	-	-	-	-	-	-	$0.27±0.01^{d}$	$0.55±0.03^{b}$
F105	44.19	n-Hexadecanoic acid	-	-	-	$1.30±0.11^{a}$	-	-	-	-	-	-	$0.39±0.01^{b}$	-
		**Furans**	**$5.22±0.13^{f}$**	**$13.56±0.18^{bc}$**	**$7.41±0.25^{e}$**	**$10.64±0.75^{d}$**	**$20.19±0.24^{a}$**	**$6.21±0.08^{f}$**	**$3.00±0.03^{g}$**	**$5.56±0.29^{f}$**	**$20.50±0.35^{a}$**	**$14.53±0.05^{b}$**	**$6.31±0.31^{ef}$**	**$12.82±0.75^{c}$**
F106	9.87	Furan, 2-pentyl-	$5.22±0.13^{g}$	$12.15±0.21^{c}$	$6.41±0.20^{f}$	$9.56±0.75^{e}$	$20.19±0.24^{a}$	$5.77±0.09^{fg}$	$2.66±0.00^{i}$	$4.07±0.27^{h}$	$20.50±0.35^{a}$	$13.43±0.04^{b}$	$5.27±0.26^{g}$	$10.96±0.70^{d}$
F107	11.93	trans-2-(2-Pentenyl)furan	-	$0.68±0.01^{c}$	$0.43±0.02^{e}$	$0.48±0.01^{d}$	-	-	-	-	-	$0.71±0.01^{b}$	$0.33±0.01^{f}$	$0.89±0.02^{a}$
F108	19.65	2,2'-Bifuran	-	-	-	-	-	-	-	-	-	-	$0.16±0.01^{b}$	$0.28±0.03^{a}$
F109	23.49	Furan, tetrahydro-2,2-dimethyl-5-(1-methylethyl)-	-	-	-	-	-	-	-	$0.67±0.03^{a}$	-	-	-	-
F110	28.76	Furan,2,2'-(1,2-ethenediyl)bis-, (E)-	-	$0.72±0.03^{b}$	$0.57±0.04^{c}$	$0.60±0.01^{c}$	-	$0.44±0.01^{d}$	$0.34±0.03^{e}$	$0.83±0.02^{a}$	-	$0.38±0.00^{de}$	$0.56±0.02^{c}$	$0.70±0.06^{b}$
		**Pyrazines**	**-**	**$1.68±0.02^{d}$**	**$4.92±0.05^{b}$**	**$10.03±0.83^{a}$**	**-**	**$4.18±0.14b^{c}$**	**$9.27±0.41^{a}$**	**$1.93±0.08^{d}$**	**-**	**$0.65±0.00^{e}$**	**$0.27±0.01^{e}$**	**$3.79±0.02^{c}$**
F111	9.73	Pyrazine	-	-	-	-	-	$0.25±0.02^{a}$	-	-	-	-	-	-
F112	11.24	Pyrazine, methyl-	-	$0.61±0.01^{b}$	$0.35±0.02^{cd}$	$0.40±0.02^{c}$	-	$1.24±0.10^{a}$	$0.57±0.05^{b}$	-	-	$0.27±0.01^{de}$	$0.16±0.00^{e}$	$0.26±0.02^{de}$
F113	12.79	Pyrazine, 2,5-dimethyl	-	-	$3.73±0.02^{b}$	$8.31±0.90^{a}$	-	-	$7.61±0.50^{a}$	-	-	-	-	$2.96±0.02^{b}$
F114	15.07	Pyrazine, trimethyl-	-	$0.47±0.02^{c}$	$0.28±0.02^{d}$	-	-	$1.51±0.01^{a}$	$0.25±0.03^{d}$	$1.12±0.04^{b}$	-	-	-	-
F115	18.22	Pyrazine, 2,5-dimethyl-3-(2-methylpropyl)-	-	-	-	$0.31±0.02^{a}$	-	-	-	-	-	-	-	$0.15±0.02^{b}$
F116	21.34	2,3-Dimethyl-5-isopentylpyrazine	-	-	-	-	-	-	-	$0.81±0.04^{a}$	-	-	-	-
F117	21.36	Pyrazine,2,5-dimethyl-3-(3-methylbutyl)-	-	-	$0.29±0.02^{d}$	$0.54±0.01^{c}$	-	$0.72±0.02^{a}$	$0.62±0.02^{b}$	-	-	-	-	-
F118	22.01	Pyrazine,2-methyl-5-propyl-	-	-	-	-	-	-	-	-	-	0.38±0.01a	-	-
F119	22.02	2-Butyl-3-methylpyrazine	-	$0.59±0.01^{a}$	$0.26±0.02^{c}$	$0.47±0.02^{b}$	-	$0.47±0.00^{b}$	$0.22±0.00^{c}$	-	-	-	$0.12±0.01^{d}$	$0.42±0.05^{b}$

		**Pyrrole**	**-**	**1.30±0.08^{a}**	**0.77±0.10^{de}**	**0.87±0.03^{cd}**	**-**	**0.99±0.06^{bc}**	**0.63±0.02^{ef}**	**1.38±0.06^{a}**	**-**	**0.53±0.00^{f}**	**0.55±0.01^{f}**	**1.06±0.13^{b}**
F120	24.93	1H-Pyrrole,1-(2-furanylmethyl)-	-	1.30±0.08^{a}	0.53±0.08^{cd}	0.52±0.01^{cd}	-	0.75±0.06^{b}	0.41±0.01^{d}	0.70±0.03^{bc}	-	0.53±0.00^{cd}	0.37±0.01^{d}	0.69±0.16^{bc}
F121	28.86	1H-Pyrrole-2-carboxaldehyde	-	-	0.24±0.02^{c}	0.34±0.03^{b}	-	0.25±0.00^{c}	0.21±0.02^{cd}	0.67±0.03^{a}	-	-	0.18±0.01^{d}	0.37±0.03^{b}
		**Disulfides**	**-**	**2.45±0.13^{b}**	**1.22±0.02^{d}**	**2.44±0.15^{b}**	**-**	**2.92±0.01^{a}**	**1.03±0.05^{de}**	**2.40±0.13^{b}**	**-**	**1.17±0.01^{d}**	**0.90±0.06^{e}**	**1.58±0.11^{c}**
F122	5.86	Disulfide, dimethyl	-	0.83±0.01^{a}	0.44±0.01^{b}	0.74±0.07^{a}	-	0.72±0.06^{a}	0.31±0.02^{c}	0.82±0.08^{a}	-	-	0.24±0.02^{c}	0.54±0.04^{b}
F123	13.96	Dimethyl trisulfide	-	1.63±0.12^{b}	0.78±0.01^{e}	1.40±0.06^{c}	-	2.20±0.06^{a}	0.72±0.03^{e}	1.58±0.05^{b}	-	1.17±0.01^{d}	0.41±0.03^{f}	1.05±0.07^{d}
F124	18.03	Trisulfide, dipropyl	-	-	-	0.31±0.02^{a}	-	-	-	-	-	-	-	-
F125	21.73	Bis(2-furfuryl)disulfide	-	-	-	-	-	-	-	-	-	-	0.25±0.02^{a}	-
		**Alkanes**	**0.56±0.02^{f}**	**3.09±0.18^{c}**	**0.17±0.01^{g}**	**2.90±0.07^{c}**	**1.19±0.06^{de}**	**3.53±0.10^{b}**	**-**	**7.46±0.15^{a}**	**1.47±0.17^{d}**	**0.92±0.00^{e}**	**-**	**-**
F126	10.92	1,2,3,4-tetramethyl-Cyclobutane	-	-	-	-	-	-	-	1.20±0.07^{a}	-	-	-	-
F127	20.83	Cyclohexane,1-methyl-4-(1-methylethylidene)-	-	3.09±0.18^{c}	0.17±0.01^{d}	2.90±0.07^{c}	-	3.53±0.10^{b}	-	4.52±0.00^{a}	-	-	-	-
F128	21.43	1-(ethenylthio)-Octane	-	-	-	-	-	-	-	1.53±0.07^{a}	-	-	-	-
F129	21.92	1,1-dimethyl-2-(2-propenyl)-Cyclopropane	0.56±0.02^{c}	-	-	-	1.19±0.06^{b}	-	-	0.21±0.00^{d}	1.47±0.17^{a}	-	-	-
F130	33.37	erythro-9,10-Dibromopentacosane	-	-	-	-	-	-	-	-	-	0.92±0.00^{a}	-	-
		**Alkenes**	**4.36±0.23^{c}**	**4.72±0.02^{c}**	**4.24±0.01^{cd}**	**7.10±0.01^{a}**	**2.59±0.13^{e}**	**4.44±0.05^{c}**	**2.70±0.07^{e}**	**6.25±0.03^{b}**	**3.07±0.46^{e}**	**3.76±0.04^{d}**	**3.07±0.14^{e}**	**6.77±0.00^{a}**
F131	6.17	Styrene	-	-	0.09±0.01^{a}	-	-	-	-	-	-	-	-	-
F132	13.64	1-Heptene, 5-methyl-	-	0.32±0.01^{d}	-	1.11±0.03^{a}	-	0.40±0.04^{c}	-	-	-	0.23±0.01^{e}	-	0.71±0.05^{b}
F133	14.57	1,3-Hexadiene, 3-ethyl-2-methyl-	-	0.27±0.01^{bc}	-	0.31±0.02^{a}	-	0.26±0.00^{bc}	-	-	-	0.27±0.01^{b}	-	0.24±0.02^{c}
F134	15.51	Ammonium acetate	-	-	1.43±0.06^{b}	2.09±0.15^{a}	-	-	-	-	-	-	-	1.47±0.02^{b}
F135	16.32	Copaene	-	-	-	-	-	-	-	-	0.61±0.00^{a}	-	-	-
F136	16.61	Guaia-1(10),11-diene	2.86±0.06^{a}	-	-	-	1.31±0.04^{b}	-	-	-	1.42±0.35^{b}	-	-	-
F137	17.36	1-U-ecene, 8-methyl-	1.50±0.17^{cde}	2.29±0.07^{a}	1.04±0.04^{gh}	1.42±0.10^{def}	1.28±0.09^{efg}	1.83±0.00^{b}	0.93±0.04^{hi}	1.67±0.10^{bcd}	1.04±0.12^{gh}	1.77±0.01^{bc}	0.68±0.04^{i}	1.17±0.09^{fgh}
F138	17.78	Benzenesulfonamide, 2-furanylmethyl-4-methyl-	-	1.84±0.07^{e}	1.67±0.11^{ef}	2.16±0.01^{cd}	-	1.94±0.09^{de}	1.77±0.03^{ef}	4.58±0.07^{a}	-	1.49±0.01^{f}	2.40±0.18^{c}	3.17±0.18^{b}
		**Imines**	**39.80±1.39^{a}**	**-**	**-**	**-**	**32.72±0.43^{b}**	**-**	**-**	**-**	**20.31±2.25^{c}**	**-**	**-**	**-**
F139	20.05	methoxy-phenyl-Oxime	39.80±1.39^{a}	-	-	-	32.72±0.43^{b}	-	-	-	20.31±2.25^{c}	-	-	-

		**Others**	**-**	**0.17±0.02[d]**	**-**	**-**	**1.67±0.09[a]**	**0.59±0.01[b]**	**0.61±0.09[b]**	**-**	**-**	**-**	**0.19±0.01[d]**	**0.39±0.07[c]**
F140	21.54	Naphthalene	-	-	-	-	0.74±0.04[a]	-	-	-	-	-	-	-
F141	22.58	Azulene	-	-	-	-	-	0.59±0.01[a]	0.61±0.09[a]	-	-	-	0.19±0.01[c]	0.39±0.07[b]
F142	23.92	1-methyl-Naphthalene	-	-	-	-	0.93±0.05[a]	-	-	-	-	-	-	-
F143	23.95	Lilial	-	0.17±0.02[a]	-	-	-	-	-	-	-	-	-	-

Note: Ala, Alanine; Phe, Phenylalanine; Val, Valine. 120, Maillard reaction time 120 min. TFAA, total free amino acid content; UFAA, Umami free amino acid content. Values followed by different superscript lowercase letters in the same column mean statistically significant differences ($p < 0.05$).

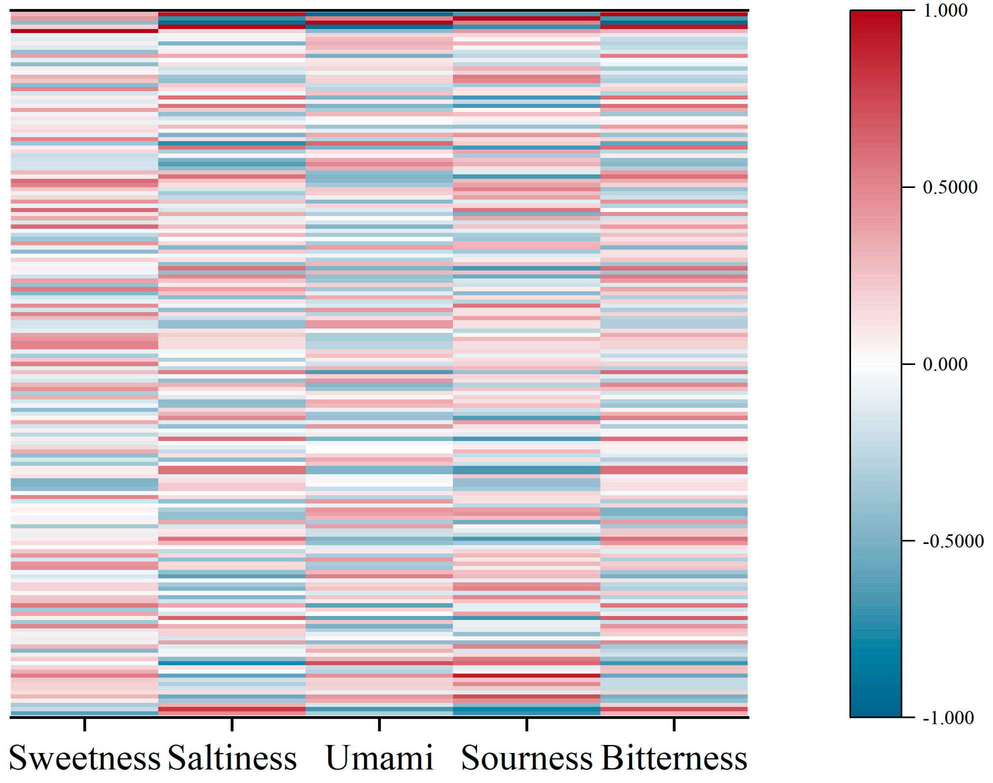

Figure S1. Pearson correlations between the molecular weight distribution, FAA contents and aroma compounds (including individual volatile organic compounds) and flavor characteristics of PPHs and their MRPs.

Table S4. Pearson correlations between the molecular weight distribution, FAAs contents, aroma compounds and flavor characteristics of PPHs and their MRPs.

Parameters	Sweetness	Saltiness	Umami	Sourness	Bitterness
< 1kDa	-0.6039*	0.4667	-0.3230	-0.7043*	0.3286
1-2 kDa	-0.2282	0.8078**	-0.6729*	-0.7885**	0.7102**
2-3 kDa	-0.3350	0.3406	-0.1575	-0.4432	0.1792
3-5 kDa	0.0833	-0.3538	0.3526	0.4740	-0.4133
5-7 kDa	0.3000	-0.5513	0.4262	0.7362**	-0.5104
>7 kDa	0.1441	0.1404	-0.0942	-0.1002	0.1869
UFAA	0.1929	-0.1719	0.1700	0.2135	-0.2254
BFAA	0.1893	-0.3049	0.1986	0.4884	-0.2244
SFAA	0.2126	-0.1836	0.1778	0.2350	-0.2340
TFAA	0.5294	-0.5917*	0.4536	0.9060***	-0.5672
Aldehydes	0.3300	0.0090	-0.2729	0.0685	0.2470
Ketones	0.1715	0.1244	-0.2360	-0.1008	0.2709
Esters	-0.0283	-0.7561**	0.7293**	0.6149*	-0.6972*
Alcohols	0.2103	-0.4356	0.3080	0.5469	-0.3973
Acids	0.0747	-0.0406	0.0613	0.1511	-0.1768
Furans	-0.4668	-0.0528	0.3343	-0.1534	-0.2610
Pyrazines	0.2976	-0.1180	0.1705	0.5191	-0.3352
Pyrrole	-0.0767	0.3858	-0.4256	-0.4289	0.4962
Disulides	0.0829	-0.1541	0.0183	0.0256	0.0153
Alkanes	-0.0486	0.1913	-0.0913	-0.3929	0.1980

Alkenes	0.1189	0.1703	-0.2992	-0.0997	0.2403
Butanal, 3-methyl-	0.5104	0.3227	-0.4940	-0.0925	0.4402
Hexanal	-0.3577	-0.0066	0.2403	-0.0885	-0.1996
2-Isopropyl-5-methylhex-2-enal	0.0682	0.6503*	-0.5804*	-0.6819*	0.6535*
Nonanal	0.3682	-0.1498	0.0129	0.3830	-0.0704
2-Octenal, (E)-	-0.3544	0.0274	0.1770	-0.0953	-0.1739
Furfural	0.5582	0.3655	-0.6060*	-0.1054	0.5636
3-Cyclohexene-1-carboxaldehyde, 1-methyl-	0.2682	-0.1605	0.0724	0.2507	-0.0761
Decanal	0.2384	-0.4683	0.2313	0.4985	-0.2776
Benzaldehyde	0.0328	-0.0314	-0.1378	-0.1533	0.2025
1H-Pyrazole-4-carboxaldehyde,1,5-dimethyl-	0.1756	-0.4805	0.2491	0.5132	-0.3040
U-ecanal	0.1743	-0.3348	0.1905	0.4705	-0.2582
Benzeneacetaldehyde	-0.0536	-0.0122	-0.0792	-0.0978	0.1171
2-Octenal, 2-butyl-	-0.1493	-0.6124*	0.5295	0.2799	-0.5083
Heptadecanal	-0.0461	-0.4135	0.2983	0.2551	-0.3710
Benzaldehyde,4-(1-methylethyl)-	0.4621	0.1637	-0.3666	0.0809	0.2483
Propanal, 2-methyl-3-phenyl-	0.4377	0.1485	-0.3242	0.2931	0.1983
Benzaldehyde,2,4-dimethyl-	-0.1423	-0.4188	0.4185	0.1067	-0.3036
2-Methyl-3(2-furyl)acrolein	0.4377	0.1485	-0.3242	0.2931	0.1983
(2-Methyl-cyclohex-2-enylidene)-acetaldehyde	0.2344	-0.2382	0.0811	0.1808	-0.1002
Benzeneacetaldehyde,.alpha.-ethylidene-	-0.0203	0.0092	-0.1108	-0.0698	0.1398
Benzeneacetaldehyde,.alpha.-(2-methylpropylidene)-	0.1506	0.3144	-0.4170	-0.3532	0.4429
trans-U-ec-4-enal	0.0691	0.5821	-0.4799	-0.6684*	0.5923*
5-Methyl-2-phenyl-2-hexenal	-0.0831	0.1202	-0.1973	-0.2257	0.2259
2-Phenyl-3-(2-furyl)-propenal	0.0763	0.1038	-0.1993	-0.0852	0.2243
2-Heptanone	-0.3417	-0.1430	0.3978	0.0378	-0.3469

3-Hexen-2-one, 5-methyl-	0.0456	0.3735	-0.3260	-0.5224	0.4012
2-Butanone,1-(2-furanyl)-3-methyl-	-0.0461	-0.4135	0.2983	0.2551	-0.3710
2,3-Octanedione	0.0129	-0.4020	0.3824	0.4448	-0.4951
2-Octanone	0.0635	-0.3114	0.4287	0.3978	-0.4979
2-Propanone,1-hydroxy-	0.0176	0.0201	-0.0927	-0.2431	0.1610
3-Octanone,2-methyl-	-0.1612	-0.4135	0.4228	0.1160	-0.3126
2,5-Hexanedione	0.5068	0.1325	-0.2620	0.1286	0.1837
2,5-Octanedione	0.0042	0.0082	0.0932	0.1720	-0.0233
5-Hepten-2-one, 6-methyl-	-0.4377	0.2128	-0.2153	-0.3521	0.1253
2-Nonanone	-0.4805	0.2208	-0.0184	-0.4167	0.1355
3-Octen-2-one	-0.4990	0.1276	0.0270	-0.4028	0.1094
2-Nonen-4-one	0.1127	-0.0956	-0.0285	0.2392	-0.0462
3-Heptanone, 2-methyl-	0.0691	0.5821*	-0.4799	-0.6684*	0.5923*
Ethanone, 1-(2-furanyl)-	0.0691	0.5821*	-0.4799	-0.6684*	0.5923*
trans-3-Nonen-2-one	-0.3535	-0.0391	0.2439	-0.1949	-0.1337
2-Butanone, 3,3-dimethyl-	-0.1612	-0.4296	0.3450	0.1160	-0.2980
3,5-Octadien-2-one, (E,E)-	-0.4088	0.1166	0.1645	-0.2151	-0.1178
Ethanone, 1-(3-methylenecyclopentyl)-	0.3440	-0.2143	-0.0003	0.2835	-0.0565
1-Propanone, 1-(2-furanyl)-	0.1678	-0.1020	0.0013	0.0648	0.0538
Acetophenone	-0.1153	-0.0242	-0.0421	-0.1078	0.0782
2-methyl-Cyclopentadecanone	0.0691	0.5821*	-0.4799	-0.6684*	0.5923*
Benzyl methyl ketone	-0.2569	-0.1153	0.0785	-0.1198	-0.0395
2(5H)-Furanone	0.0522	-0.0119	-0.0446	0.0613	0.0436
2(3H)-Furanone,5-dihydro-5-methyl-	-0.1612	-0.4135	0.4228	0.1160	-0.3126
Nona-3,5-dien-2-one	0.3462	-0.1223	-0.0973	0.4043	-0.0542
Isophorone	0.0375	0.5005	-0.3965	-0.6448*	0.5304

1,2-Propanedione, 1-phenyl	-0.1405	0.3098	-0.3788	-0.2854	0.3136
Tetrahydrocarvone	-0.4267	0.1526	-0.0748	-0.1949	0.0391
3-Ethoxy-2-methyl-2-cyclohexen-1-one	-0.0461	-0.4135	0.2983	0.2551	-0.3710
2-Sec-Butylcyclohexanone	-0.1612	-0.4296	0.3450	0.1160	-0.2980
Cyclohexanone, 2-octyl	0.3365	-0.1354	0.0303	0.1798	-0.0310
(Z)-6,10-dimethyl-5,9-U-ecadien-2-one	-0.3154	0.0314	0.1438	-0.0697	-0.1650
5,9-U-ecadien-2-one,6,10-dimethyl-	0.4544	0.1319	-0.3784	0.2435	0.2675
3-Buten-2-one,4-(2-furanyl)-	0.2990	0.3261	-0.4594	-0.2812	0.4903
Bicyclo[3.3.0]octan-2-one, 7-isopropylidene	-0.1612	-0.4135	0.4228	0.1160	-0.3126
Bicyclo[3.1.0]hex-3-en-2-one	-0.0921	-0.0442	0.1738	0.1666	-0.1083
2(1H)-Azulenone,4,5,6,7,8,8a-hexahydro-8a-methyl-	0.2604	0.5340	-0.6491*	-0.3875	0.6072*
2-Pentadecanone	-0.1071	-0.2439	0.3060	0.2782	-0.2817
2-Tridecanone	0.5397	0.0204	-0.1820	0.1718	0.1940
1,3-Dioxol-2-one,4,5-dimethyl-	0.2199	-0.2961	0.0853	0.1454	-0.0756
Solavetivone	-0.3380	-0.0015	0.2509	-0.0741	-0.2264
heptafluorobutyrate	-0.0921	-0.0442	0.1738	0.1666	-0.1083
Menthyl acetate	0.5068	0.1325	-0.2620	0.1286	0.1837
Pentafluoropropionic acid, hexadecyl ester	0.5068	0.1325	-0.2620	0.1286	0.1837
6-Bromohexanoic acid,heptyl ester	0.4377	0.1485	-0.3242	0.2931	0.1983
3-Hydroxypyridinemonoacetate	0.3916	0.2128	-0.3242	0.0274	0.3442
4-Ethylbenzoic acid,cyclopentyl ester	-0.1052	0.1245	-0.0084	-0.2559	0.1535
2-Nitrophenyl-.beta.-phenylpropionate	-0.1612	-0.4135	0.4228	0.1160	-0.3126
Isopropyl palmitate	-0.1612	-0.4135	0.4228	0.1160	-0.3126
Hexadecanoic acid,ethyl ester	0.2695	-0.1996	0.1397	0.3884	-0.2899
9-Octadecenoic acid,methyl ester, (E)-	0.5068	0.1325	-0.2620	0.1286	0.1837
11-Octadecenoic acid,methyl ester	-0.1612	-0.4135	0.4228	0.1160	-0.3126

1,2-Benzenedicarboxylic (2-methylpropyl) ester	0.4690	-0.0519	-0.1362	0.5699	-0.1156
Dibutyl phthalate	-0.0692	0.1129	-0.1966	-0.2653	0.1777
2-Hexanol, 3,4-dimethyl-	-0.1612	-0.4296	0.3450	0.1160	-0.2980
1-Hexanol	-0.4607	0.2449	-0.0908	-0.4407	0.1983
3,5-Octadien-2-ol	0.5452	0.3938	-0.3429	0.0718	0.3599
1-Octen-3-ol	-0.4050	0.0820	0.1913	-0.1929	-0.1236
1,6-Octadien-3-ol,3,7-dimethyl-	0.3968	0.2331	-0.3656	-0.1442	0.4102
1-Octanol	-0.1900	0.5001	-0.4012	-0.5419	0.5403
1-Hexanol, 3-methyl-	-0.0461	-0.4135	0.2983	0.2551	-0.3710
Orcinol	0.0691	0.5821	-0.4799	-0.6684*	0.5923*
3-Nonen-1-ol, (Z)-	-0.0461	-0.4135	0.2983	0.2551	-0.3710
Cyclohexanol, 5-methyl-2-(1-methylethyl)-, acetate,	0.1794	0.1167	-0.2912	0.0673	0.2330
2-Furanmethanol	-0.0013	-0.0579	-0.0399	-0.1234	0.1220
E-11,13-Tetradecadien-1-ol	-0.4377	0.2128	-0.2153	-0.3521	0.1253
1-Nonanol	-0.0787	-0.4574	0.3917	0.3121	-0.4612
2,4-Pentadien-1-ol,3-pentyl-, (2Z)-	0.4377	0.1485	-0.3242	0.2931	0.1983
Benzyl alcohol	-0.3772	-0.0124	-0.0106	-0.3642	0.1097
1-Dodecanol	-0.2416	0.2927	-0.3435	-0.3194	0.2675
Cyclopropanol, 2,2-dimethyl-3-(2-phenylethynyl)-	-0.0325	0.0281	-0.1084	-0.0977	0.1413
p-Cymen-7-ol	0.6141*	0.2689	-0.4808	0.2333	0.4046
2-Phenyl-2,4-octadienol	-0.1278	0.0742	-0.1460	-0.1668	0.1544
1-Hexadecanol	0.3491	-0.0759	0.0032	0.3574	-0.1823
Acetic acid	0.1225	0.3508	-0.3019	-0.4952	0.4665
Hexanoic acid	0.6215*	-0.1597	-0.1154	0.5658	0.0108
Propanoic acid,2-methyl-	-0.0788	-0.0939	0.1696	0.1690	-0.2798
Octanoic acid	0.4403	0.2518	-0.4399	-0.1012	0.4406

Nonanoic acid	0.1616	-0.2432	0.0689	0.3888	-0.1845
n-Hexadecanoic acid	0.0687	-0.3447	0.1989	0.2589	-0.2646
Furan, 2-pentyl-	0.2362	-0.1583	0.2295	0.5096	-0.3835
trans-2-(2-Pentenyl)furan	0.5208	0.1359	-0.3529	0.3827	0.1905
2,2'-Bifuran	0.5971*	0.2435	-0.4598	0.2800	0.3557
Furan, tetrahydro-2,2-dimethyl-5-(1-methylethyl)-	0.0691	0.5821*	-0.4799	-0.6684*	0.5923*
Furan,2,2'-(1,2-ethenediyl)bis-, (E)-	0.3026	0.2625	-0.4214	-0.1168	0.4174
Pyrazine	-0.1612	-0.4296	0.3450	0.1160	-0.2980
Pyrazine, methyl-	-0.1666	-0.6198*	0.4878	0.3228	-0.4486
Pyrazine, 2,5-dimethyl	-0.1803	-0.4951	0.3640	0.2365	-0.4079
Pyrazine, trimethyl-	-0.2128	-0.0349	0.0637	-0.3233	0.0686
Pyrazine, 2,5-dimethyl-3-(2-methylpropyl)-	0.1576	-0.3171	0.1293	0.3720	-0.2547
2,3-Dimethyl-5-isopentylpyrazine	0.0691	0.5821*	-0.4799	-0.6684*	0.5923*
Pyrazine,2,5-dimethyl-3-(3-methylbutyl)-	-0.3762	-0.7382**	0.6186*	0.1921	-0.5810*
Pyrazine,2-methyl-5-propyl-	0.5068	0.1325	-0.2620	0.1286	0.1837
2-Butyl-3-methylpyrazine	-0.0942	-0.4804	0.3480	0.4278	-0.3769
1H-Pyrrole,1-(2-furanylmethyl)-	0.1559	0.0058	-0.0916	0.0846	0.1211
1H-Pyrrole-2-carboxaldehyde	0.1177	0.2792	-0.3841	-0.3875	0.4122
Disulfide, dimethyl	-0.0703	-0.0623	-0.0003	-0.0820	0.0497
Dimethyl trisulfide	0.1182	-0.1630	0.0226	0.0413	0.0093
Trisulfide, dipropyl	-0.0461	-0.4135	0.2983	0.2551	-0.3710
Bis(2-furfuryl)disulfide	0.3916	0.2128	-0.3242	0.0274	0.3442
1,2,3,4-tetramethyl-Cyclobutane	0.0691	0.5821*	-0.4799	-0.6684*	0.5923*
Cyclohexane,1-methyl-4-(1-methylethylidene)-	-0.1229	-0.0312	0.0687	-0.2318	0.0381
1-(ethenylthio)-Octane	0.0691	0.5821*	-0.4799	-0.6684*	0.5923*
1,1-dimethyl-2-(2-propenyl)-Cyclopropane	-0.1546	-0.0364	0.2446	0.0714	-0.2855

erythro-9,10-Dibromopentacosane	0.5068	0.1325	-0.2620	0.1286	0.1837
Styrene	-0.4377	0.2128	-0.2153	-0.3521	0.1253
1-Heptene, 5-methyl-	0.2289	-0.4086	0.1867	0.4995	-0.3068
1,3-Hexadiene, 3-ethyl-2-methyl-	0.3351	-0.3707	0.1627	0.5363	-0.2542
Ammonium acetate	-0.0298	-0.1282	-0.0605	0.1728	-0.1144
Copaene	0.0691	-0.1726	0.1738	0.3184	-0.3272
Guaia-1(10),11-diene	-0.4302	0.1183	0.1120	-0.2376	-0.0600
1-U-ecene, 8-methyl-	-0.0985	-0.0023	0.1052	-0.0692	-0.0207
Benzenesulfonamide, 2-furanylmethyl-4-methyl-	0.3860	0.3555	-0.5082	-0.2514	0.5395
methoxy-phenyl-Oxime	-0.4089	0.0844	0.1811	-0.1929	-0.1208
Naphthalene	-0.1152	-0.0602	0.2827	0.0401	-0.2250
Azulene	0.0607	-0.4863	0.3265	0.3091	-0.2680
1-methyl-Naphthalene	-0.1152	-0.0602	0.2827	0.0401	-0.2250
Lilial	-0.0921	-0.0442	0.1738	0.1666	-0.1083
Sweetness	1.0000	0.1868	-0.4335	0.4290	0.3100
Saltiness	0.1868	1.0000	-0.9170***	-0.7091**	0.9465***
Umami	-0.4335	-0.9170***	1.0000	0.5131	-0.9517***
Sourness	0.4290	-0.7091**	0.5131	1.0000	-0.6719*
Bitterness	0.3100	0.9465***	-0.9517***	-0.6719*	1.0000

Note: TFAA, total free amino acid content; SFAA: Sweet free amino acid content; BFAA: Bitter free amino acid content; UFAA, Umami free amino acid content. *: $p < 0.05$; **: $p < 0.01$; ***: $p < 0.001$. The reported p values are uncorrected for multiple comparisons.
